# Synchrotron imaging reveals diversity in archosauromorph growth strategies by the end of the early Triassic

**DOI:** 10.1016/j.isci.2026.116162

**Published:** 2026-06-03

**Authors:** Kathleen.N. Dollman, Frederick.B. Tolchard, Jonah.N. Choiniere, John Hancox, Andrew B. Heckert, Chandelé Montgomery, Valentin Buffa, Jennifer Botha

**Affiliations:** 1 European Synchrotron and Radiation Facility, Grenoble 38000, France; 2 Evolutionary Studies Institute, University of the Witwatersrand, 1 Jan Smuts Avenue, Braamfontein, Johannesburg 2000, South Africa; 3 Department of Geological and Environmental Sciences, Appalachian State University, ASU Box 32067, Boone, NC 28608, USA; 4 GENUS: DSI-NRF Centre of Excellence in Palaeosciences, University of the Witwatersrand, Johannesburg 2000, South Africa; 5 National Museum, Bloemfontein 9301, South Africa; 6 Department of Paleontology, University of Zurich, Zurich 8006, Switzerland

**Keywords:** X-ray optics, biological sciences, paleobiology, palaeohistology

## Abstract

Archosauromorphs are a diverse clade of vertebrates that originated in the Early Triassic and persist today as crown birds (ornithodirans) and crocodilians (pseudosuchians). They exhibit markedly different life-history strategies, with ornithodirans characterized by rapid growth, and more crown-ward pseudosuchians with slower growth rates. Understanding how these patterns emerged requires further insight into growth dynamics among early archosauromorphs. We use synchrotron X-ray micro-computed tomography to investigate the bone microstructure in an assemblage of archosauromorphs from the Early Triassic locality Driefontein Farm 11 in the Olenekian-Anisian *Langbergia-Garjainia* subzone of the *Cynognathus* zone in the main Karoo Basin of South Africa. Our findings reveal that growth strategies among Early Triassic archosauromorphs already encompass both rapid and slow growth rates. We further deduce that the diversity in growth strategies observed in crown-ward archosaur lineages were not lineage-specific derived characteristics but rather inherited growth traits already present among their Early Triassic members.

## Highlights

• Synchrotron micro-CT of Early Triassic archosauromorph bones reveals diverse growth strategies
• Results suggest fast growth is not plesiomorphic in Archosauromorpha
• Results suggest at least 9 distinct Early Triassic archosauromorph taxa
• Driefontein archosauromorph diversity is geographically and temporally localized

## Graphical abstract

**Figure:**
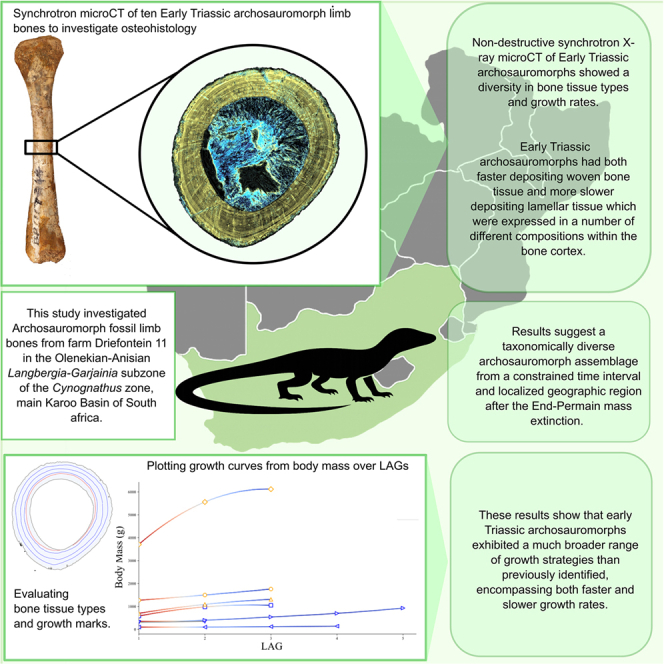

## Introduction

The evolution of growth, the biological process of increasing in size through maturity, remains a central question in vertebrate biology because growth strategies are closely linked to key life history traits such as adult body size,^1^ immune resilience,^2^ maturation,^3^ and basal metabolic rates.^4^ Our knowledge of growth strategies among more crown-ward vertebrate groups is advancing, particularly among mammals^5^^,^^6^ and across the theropod-to-bird transition.^7^^,^^8^^,^^9^ However, a major gap remains in our understanding of how growth strategies were expressed among the various stem lineages across vertebrates, information which is crucial for reconstructing the ancestral states of life history traits.

Archosauromorphs, the taxonomically expansive clade that includes Pseudosuchia, Ornithodira, and their closest fossil relatives, first diversified in the Early Triassic (∼252 Ma). Later-evolving archosaurian archosauromorphs express a divergence in evolutionary trajectories of growth strategies, with general deceleration of growth rates in pseudosuchians contrasting with acceleration of growth rates of ornithodirans.^7^^,^^8^^,^^10^^,^^11^ These salient differences in growth strategies between pseudosuchian and ornithodiran lineages, which are exemplified by the stark differences between living birds and crocodilians, suggest a deeper divergence in their evolutionary history, potentially among their stem members, the archosauromorphs. Our current understanding of stem archosauromorph growth strategies relies on a limited number of studies, which suggest that faster growth rates are the ancestral condition for Archosauromorpha.^12^^,^^13^^,^^14^ However, growth rates are often correlated with body size, with faster sustained growth rates often underlying larger maximum achievable adult body sizes.^7^^,^^8^^,^^15^^,^^16^^,^^17^^,^^18^^,^^19^^,^^20^ Therefore, the true diversity of growth strategies in archosauromorphs may be obscured by a historic sampling bias toward larger bodied forms.^12^^,^^14^^,^^21^ Existing studies of mid-late Triassic archosaurs have revealed a surprising diversity in bone tissue microstructure and growth strategies^22^ but this variability has largely been attributed to geographic and environmental differences.^12^^,^^13^^,^^23^^,^^24^ However, comparatively little is known about growth strategies among Early Triassic archosauromorphs. In particular, it remains unclear whether faster growth strategies or more diverse growth strategies are more prevalent among archosauromorphs at this time.

In the fossil record, bone tissue microstructures preserve a wealth of life-history information that is otherwise completely inaccessible. Bone tissue types can reveal different growth rates; for example, the disorganized matrix of woven-fibred bone is associated with rapid growth, whereas highly regular lamellar bone reflects slower growth.^25^ Additional osteohistological features, including vascularity, cortical thickness, and the presence and spacing of annual growth marks such as lines of arrested growth (LAGs), provide further insight into the tempo and seasonality of growth. LAGs, which generally correspond to annual pauses in growth, are widely used to estimate individual age in years^26^ and offer the only direct proxy for annualized body-mass gain in extinct species.^7^^,^^27^ Conventional access to bone histology requires destructive thin-sectioning of limb bones and examination of thin sections under transmitted light microscopy. Despite methodological advances, sampling of physical thin-sections of the osteohistology of small-bodied vertebrates faces a number of challenges, including loss of fossil material with destructive sampling, sectioning imprecision, and interpretive ambiguity.^25^^,^^28^^,^^29^^,^^30^^,^^31^^,^^32^^,^^33^ Advances in synchrotron X-ray micro-computed tomography (SR-μCT) allow non-destructive visualization of bone histology at sub-micron resolution, even in some of the smallest vertebrates.^34^ Although SR-μCT osteohistology presents its own challenges, including data management and rendering, data interpretation, imaging artifacts, and the inefficiency of visualizing fiber orientations, its benefits include the ability to interpret bone tissue microstructures in 3D^34^^,^^35^ and to rapidly accumulate large sample sets for quantitative tissue analysis.^36^

This study addresses this gap in scientific knowledge of growth strategies among Early Triassic archosauromorphs by applying SR-μCT to non-destructively investigate the osteohistology of several small-bodied archosauromorphs from the farm Driefontein 11, a fossil lagerstätte in South Africa. Strata at farm Driefontein 11,^37^^,^^38^^,^^39^ were deposited in a geographically restricted riparian habitat during the late Olenekian-Anisian period, some 247 million years ago. The age of the Driefontein 11 locality, situated within Subzone A of the *Cynognathus* Assemblage Zone in the Karoo Basin, has traditionally been assigned a late Olenekian age but uncertainties with global vertebrate biostratigraphic correlations indicate an Anisian age is also possible.^37^^,^^40^ Accordingly, Driefontein 11 is considered late Olenekian-Anisian in age for the purposes of this study. Future geochronological work on the Triassic Karoo deposits will be essential for more definitive age constraints.

Our results reveal a surprising diversity of growth strategies within the Driefontein archosauromorph faunal assemblage, suggesting that Triassic archosauromorphs already exhibited diverse growth patterns relatively soon after the end-Permian mass extinction, and potentially, that faster growth rates are not the plesiomorphic condition for the group.

## Results

### The osteohistology of the archosauromorphs

#### BP/1/6232

**Figure 1:**
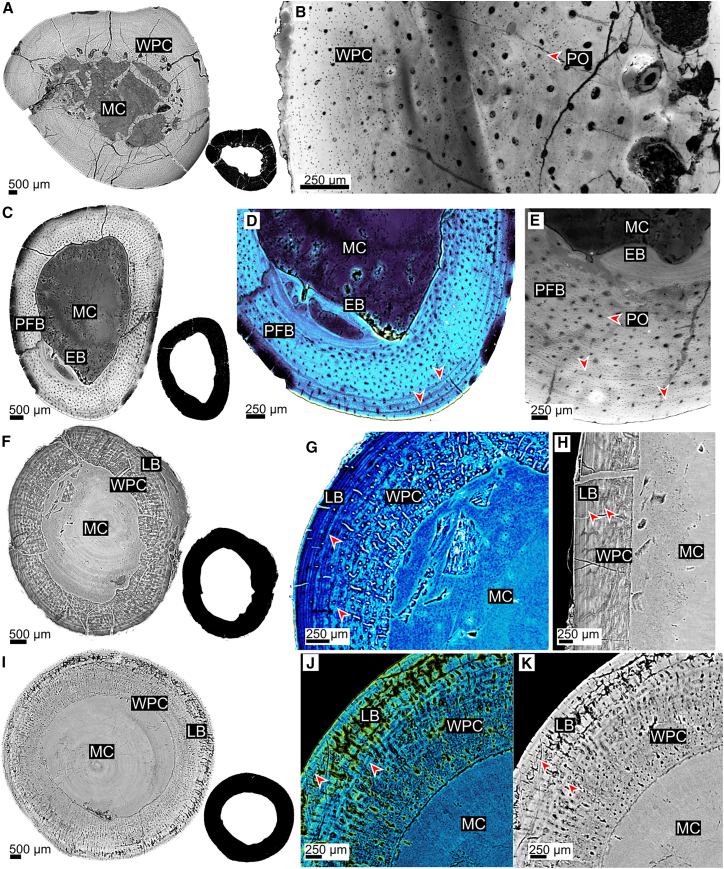
Bone tissue microstructure Bone tissue microstructure of (A and B) BP/1/6232, (C–E) BP/1/7114, (F–H) BP/1/9030, and (I–K) BP/1/8854 (A) Shows a 2 μm rendering of the complete cross-section of BP/1/6232 with a binary version of the cortex. (B) Images at 0.7 μm isotropic voxel size of the cortex of BP/1/6232 indicating the presence of primary osteons. There is the presence of imaging artifacts such as color gradient differences and rings that we have excluded and are not related to bone tissue structures and LAGs. (C) Shows a 2 μm rendering of the complete cross-section of BP/1/7114 and a binary version of the cortex, with (D) optimized colored histogram to enhance contrast of growth lines, and (E) and image of the cortex at 0.7 μm isotropic voxel size showing a magnified region of the cortex indicating the presence of primary osteons. (F) Shows a 2 μm rendering of the complete cross-section of BP/1/9030 and a binary version of the cortex, with (G) optimized colored histogram to enhance contrast of growth lines, and H) a vertical axis section of the cortex of BP/1/9030 to illustrate continuity of LAGs through the cortex. (I) Shows a 2 μm rendering of the complete cross-section of BP/1/8854 and a binary version of the cortex, with (J) an optimized colored histogram rendered with a thick slab of minimal values of a section of the cortex to enhance contrast of growth lines, and (K) the same section of the cortex rendered with gray values and with an average thick slab to highlight changes in bone tissue across the cortex. LAGS and labeled primary osteons are indicated by red arrows. MC, medullary cavity; LB, lamellar bone; PO, primary osteon; WPC, woven-parallel complex; WB, woven bone. Scale bars: 500 μm in (A), (C), (F), and (I) and 250 μm in (B), (D), (E), (G), (H), (J), and (K).

The humerus of BP/1/6232 is referred to Archosauromorpha because it lacks an entepicondylar foramen and presents an incipient ulnar condyle.^41^ BP/1/6232 is one of the larger specimens in the dataset, with an estimated maximum body mass of 16.09 kg (Table 1). BP/1/6232 shows a proportionally thicker cortex and a large medullary cavity, with minimal secondary remodeling (Figures 1A and 1B). The section was taken closer to the distal end and thus a few trabeculae are visible traversing the medullary cavity. The cortex is moderately vascularized, featuring longitudinal simple canals. Tiny patches of woven bone are present throughout the cortex and a few primary osteons in a woven-parallel complex (WPC) are present in the innermost cortex (Figure 1B). There are no observable lines of arrested growth (LAGs), the rings in the inner and mid cortex (Figures 1A and 1B) are a result of imaging artifacts. The lack of any observable LAGs suggests that this individual was at an early ontogenetic stage.

**Table 1:** Presents a summary of the bone osteohistology

Specimen Number	Identification	Limb element	Cortical thickness (mm)	Cortical thickness (%)	Circumference measurement (mm)	Body mass estimate (kg)	Bone tissue type inner cortex	Bone tissue type outer cortex	LAG	Vascularity	Primary/secondary osteon	Osteocyte orientation
BP/1/6232	Archosauromorpha	complete humerus	2.7	34.4	37.02	8.57–16.09	woven-parallel	woven-parallel	0	longitudinal	primary	random
BP/1/7114	Archosauromorpha	complete humerus	1.02	18.4	18.26	1.33–2.33	woven-parallel	woven-parallel	2	longitudinal	primary	random
BP/1/5661	Archosauromorpha	complete ulna indet.	2.17	35.3	27.53	N/A	woven-parallel	woven-parallel/lamellar	2	longitudinal/radial	primary	random
BP/1/8854	Archosauromorpha	proximal femur	1.99	25.6	30.95	4.33–8.67	woven-parallel	lamellar	2	longitudinal radial	primary	random
BP/1/9546	Archosauromorpha	proximal femur	0.69	21.4	11.32	0.26–0.47	woven-parallel	woven-parallel	1	longitudinal	primary	random
BP/1/7344	Archosauromorpha	complete femur	0.99	19.8	16.69	0.77–1.45	lamellar/parallel	lamellar/parallel	2	longitudinal	primary	parallel
BP/1/9552	Archosauromorpha	distal femur	0.92	21.1	15.96	0.62–1.16	lamellar	lamellar	4	longitudinal	primary	parallel
BP/1/9549	Archosauromorpha	proximal femur	0.41	16.6	8.21	0.11–0.18	Lamellar	lamellar	3	longitudinal	primary	parallel
BP/1/9030	Archosauromorpha	complete femur	1.08	20.4	18.03	0.96–1.81	woven-parallel	lamellar	2	longitudinal radial	absent	?

Bone osteohistology includes cortical thickness, limb bone circumference measurements, estimated body mass (calculated only for femora and humeri, and representing the lower and upper 95 percentile estimate values), general bone tissue type (woven, parallel or lamellar fiber complex), observed number of LAGs, vascularization orientation, the presence of primary or secondary osteons, and the primary orientation of osteocytes.

#### BP/1/7114

The proximal portion of a humerus, BP/1/7114, is referred to Archosauromorpha based on the presence of a prominent conical crest positioned dorsal to the deltopectoral crest, which closely resembles *Prolacerta broomi* (BP/1/2675), as well as the allokotosaurs *Malerisaurus*,^42^ *Pamelaria*,^41^ and *Trilophosaurus*.^43^ Furthermore, all femora that preserve the proximal surface (BP/1/9030, BP/1/7344, BP/1/9549, BP/1/8854, BP/1/9546) can be referred to the clade including Archosauriformes and several subclades of non-archosauriform archosauromorphs based on the presence of a partially ossified proximal articular surface with a circular concavity.^41^ The humerus of BP/1/7114 has a comparatively thinner and compact cortex, which surrounds a large and open medullary cavity (Figures 1C–1E). BP/1/7114 is a relatively mid-sized individual among the sample set with an estimated maximum body mass of 2.33 kg (Table 1). Along a section of the surface of the inner bone cortex within the medullary cavity, BP/1/7114 presents thick endosteal bone (Figures 1C–1E). The cortex of BP/1/7114 consists of PFB, with simple, longitudinal vascular canals, and a few identifiable primary osteons (Figure 1E). The size and abundance of the simple canals decreases in the outer cortex and contains two fairly closely spaced LAGs (Figures 1D and 1E). The presence of lamellae toward the outer cortex suggests that BP/1/7114 represents a maturing individual. However, the absence of an outer circumferential lamellae (OCL), a layer of closely packed lamellae near the periosteal surface that signals a slowdown or cessation of growth at skeletal maturity, indicates that BP/1/7114 had not yet reached skeletal maturity.

#### BP/1/9030

BP/1/9030 exhibits a relatively highly vascularized WPC in the inner cortex (Figures 1F–1H). The vascular canals range from longitudinal to short radial canals. BP/1/9030, resembling BP/1/7114, has a thinner cortex around a wide medullary cavity. Additionally, BP/1/9030 is a mid-sized individual with respect to the sample set, with an estimated body size of 1.31 kg (Table 1) The bone tissue transitions from WPC in the inner cortex into poorly vascularized lamellar bone with only a few longitudinally oriented simple canals in the outer cortex (numerous lamellae can be seen from the mid- to outer-cortex). We identify at least two, and possibly as many as five (Figures 1G and 1H), closely spaced LAGs in the outer cortex but the exact number is difficult to confirm among the numerous lamellae. A few short radial vascular canals in the outermost cortex suggests that although the pace of growth has decreased in this region, there is no confirmable OCL. However, the observable shift to lamellar bone (LB) in the outer cortex shows that BP/1/9030 was not at an early ontogenetic stage.

#### BP/1/8854

The femur of BP/1/8854 exhibits a relatively thin cortex with a large open medullary cavity (Figures 1I–1K). The bone cortex appears to be heavily diagenetically altered and so bone tissue features are difficult to confirm. The inner cortex is highly vascularized with simple longitudinal canals that are linked by transverse anastomoses, which are revealed in vertical axis section (Figures 1J and 1K). Many of the mid-cortical canals extend as short radial canals. Vascularization decreases toward the outer cortex with a clear change in bone tissue type. The inner and mid-cortex likely comprises WPC (Figures 1J and 1K). There are two LAGs visible in the outer cortex within the LB (Figures 1J and 1K). Although difficult to discern, there is no observable OCL along the outermost cortex, and so it is unlikely this individual had reached complete skeletal maturity but the presence of LB bone suggests this individual had passed the early ontogenetic growth stage.

#### BP/1/9546

**Figure 2:**
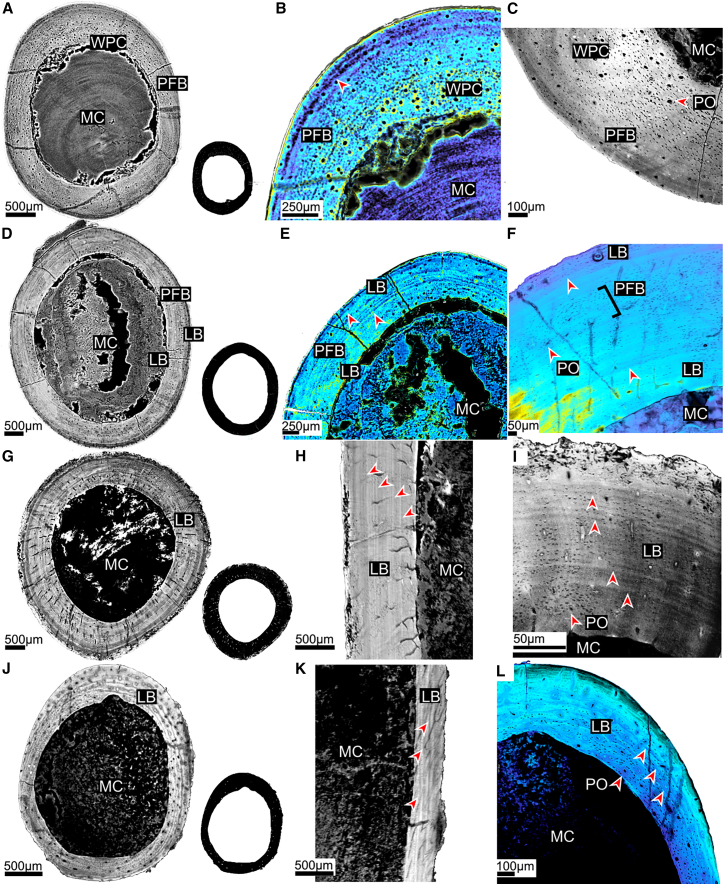
Bone tissue microstructure Bone tissue microstructure of (A–C) BP/1/9546; (D–F) BP/1/7344; (G–I) BP/1/9552, and (J–L) BP/1/9549. (A) Shows a 2 μm rendering of the complete cross-section of BP/1/9546 and a binary version of the cortex, with (B) optimized colored histogram to enhance contrast of growth lines, and (C) and image of the cortex at 0.7 μm isotropic voxel size showing a magnified region of the cortex indicating the presence of primary osteons. (D) Shows a 2 μm rendering of the complete cross-section of BP/1/7344 and a binary version of the cortex, with (E) optimized colored histogram to enhance contrast of growth lines, and (F) and image of the cortex at 0.7 μm isotropic voxel size showing a magnified region of the cortex showing lamellae and osteocyte orientation. (G) Shows a 2 μm rendering of the complete cross-section of BP/1/9552 and a binary version of the cortex, with (H) vertical axis section showing the continuity of growth marks, and (I) and image of the cortex at 0.7 μm isotropic voxel size showing a magnified region of the cortex showing lamellae and primary osteons. (J) shows a 2 μm rendering of the complete cross-section of BP/1/9549 and a binary version of the cortex, with (K) a vertical axis section showing the continuity of growth marks, and (L) and image of the cortex at 0.7 μm isotropic voxel size showing a magnified region of the cortex showing lamellae and primary osteons. Scale bars: 500 μm in (A), (D), (G), (H), (J), and (K); 250 μm in (B) and (E); 100 μm in (C) and (L); and 50 μm in (F) and (I).

The proximal portion of femur BP/1/9546 exhibits a comparatively thinner compact cortex, with a large, open medullary cavity. BP/1/9546 is one of the smaller individuals sampled with a maximum body mass estimated at 0.47 kg (Table 1). The inner to mid-cortex contains tiny patches of woven bone but most of the cortex consists of PFB, which becomes poorly vascularized toward the outer cortex (Figures 2A–2C). There are primary osteons along the inner to mid cortex (Figure 2C). The outer cortex has simple, longitudinal vascular canals. There is one visible growth mark in the outer cortex (Figures 2B and 2C). The vascular canals extend to the outermost cortex and there is no observable OCL, and so BP/1/9546 is not considered skeletally mature but the shift to slower growth suggests BP/1/9546 is a small bodied subadult.

#### BP/1/7344

The femur of BP/1/7344 has a relatively thin compact cortex (Figures 2D–2F), similar by percentage to BP/1/7114, BP/1/9546, BP/1/9552, and BP/1/9030 (Table 1). The estimated maximum body mass of BP/1/7344 is 1.45 kg. The inner and outer cortex contains small, flat osteocyte lacunae arranged mostly in parallel indicating LB (Figure 2F), although there are patches of more disorganized osteocyte lacunae suggesting the presence of PFB (Figure 2F). The bone tissue is poorly vascularized, with longitudinally oriented simple canals and several thin radial canals throughout the cortex. There are two confirmed LAGs, the first is observable in the inner, mid-cortex, and a second closer positioned closer to the outer cortex (Figure 2F). There are additionally three LAGs on the other cortex, which are closely spaced but do not appear to be continuous in vertical axis, and so may instead represent a triple LAG, which are three closely spaced LAGs (Figure 2E). Furthermore, there is no indication of an OCL along the outer cortex, and so, although this individual had grown over at least two growth seasons, it is considered a subadult.

#### BP/1/9552

The distal portion of the femur of BP/1/9552 has sub-rectangular margins and an epiphysis that is markedly wider than the diaphysis. This morphology strongly resembles the distal femur of a referred specimen of *Prolacerta broomi* (BP/1/2675); however, the osteohistology of BP/1/9552 is distinct from *Prolacerta*,^14^ so based on this BP/1/9552 can be considered an archosauromorph but it is unlikely to be referred to *Prolacerta*. BP/1/9552 has a thin, compact cortex with a wide, open medullary cavity and no evidence of secondary remodeling (Figure 2G). The maximum body mass of BP/1/9552 is estimated at 1.16 kg (Table 1), which is only marginally less than that of BP/1/7344. The cortex is comprised of numerous lamellae and the relatively poorly vascularized tissue contains longitudinal and radial simple canals through the cortex, indicating predominantly LB (Figures 2G–2I). The LB is distinct from the osteohistology described for *Prolacerta*, which is WB and PFB.^14^ There is a decrease in vascularization toward the subperiosteal surface. There are at least four visible LAGs, which are continuous in vertical axis (Figure 2H) but the lamellae make it difficult to distinguish the LAGs from lamellae, and so there may be more. There is an indication of an OCL on the outermost edge of the cortex, and so this individual could have achieved full maturity at time of death but the edge is heavily eroded and so the presence of an OCL cannot be confirmed.

#### BP/1/9549

BP/1/9549 is the smallest of the sampled archosauromorphs, with an estimated maximum body mass of just 0.18 kg (Table 1). The bone cortex is also the proportionately thinnest of the samples, comprising only 16.6% of the total cross-section (Table 1). The thin compact cortex surrounds a large open medullary cavity (Figure 2J). The bone wall is comprised of LB as most of the osteocyte lacunae are aligned in parallel (Figures 2K and 2L). The vascular canals are simple and longitudinally orientated. There are three closely spaced LAGs that are continuous along the cortex in vertical axis section (Figures 2J and 2K). However, in transverse view, the LAGs are interrupted along the one surface, most likely due to erosion during preservation, suggesting that these are annual growth marks and not three closely spaced growth marks (triple LAG). Along the outer cortex, there is no indication of an OCL, and so this individual was not completely skeletally mature but because it had already passed three seasons, it is likely that BP/1/9549 was at least a subadult.

## Discussion

Our findings greatly expand our understanding of Early Triassic archosauromorph diversity by revealing pronounced taxonomic diversity and a previously unrecognized breadth of growth strategies within a localized archosauromorph assemblage shortly after the end-Permian mass extinction (EPME). Globally, there are fewer than 20 named archosauromorph taxa known from the Early Triassic period.^41^ Three of these taxa come from the well-constrained main Karoo Basin, including *Proterosuchus* and *Prolacerta* from the *Lystrosaurus* Assemblage zone in the underlying Induan, Katberg formation,^44^^,^^45^ and *Garjainia*, which was also discovered from farm Driefontein 11 in the Olenekian-Anisian *Langbergia*-*Garjainia* subzone of the *Cynognathus* zone.^21^ Other relevant archosauromorphs from the main Karoo Basin include *Erythrosuchus* and *Euparkeria* from the overlying middle Triassic Anisian/Ladinian *Trirachodon*-*Kannemeyeria* subzone of the *Cynognathus* zone.^21^^,^^37^

The morphological and osteohistological differences described here point to as many as nine distinct archosauromorph taxa. The observation that farm Driefontein 11 hosts a diverse archosauromorph assemblage is further supported from earlier observations from diapsid dental assemblage, where seven distinct dental morphotypes were identified.^46^ Understanding the timing of the radiation of archosauromorphs during the Triassic period is crucial to understanding their recovery from the EPME, since they filled many niches previously dominated by therapsids or parareptiles during the Late Permian. However, much debate still surrounds the timing and tempo of their recovery and radiation.^46^^,^^47^^,^^48^^,^^49^^,^^50^ Our results demonstrate that a diverse archosauromorph assemblage with key differences in their growth strategies was emplaced within five million years of the EPME, and support mounting evidence drawn from the ichnological record,^51^^,^^52^ that the earliest archosauromorph radiation was already underway early in the Triassic. Furthermore, all archosauromorph fossils were recovered from a narrowly constrained temporal interval and a localized geographic setting, indicating the presence of complex and diverse archosauromorph assemblages not long after the EPME.

Although the superficial anatomy of the new specimens presented here precludes confident assignment to specific archosauromorph subclades, variation in bone tissue microstructure clearly distinguishes these specimens as independent archosauromorph taxa. Arguably, the taxonomic diversity may be inflated because of inherent plasticity of vertebrate growth dynamics, which is reflected in bone tissue histology.^16^^,^^31^^,^^33^^,^^53^^,^^54^^,^^55^ However, growth plasticity within species, and even among appendicular elements of a single individual, primarily manifests as stochastic placing of cyclical growth marks and variations in duration of faster and slower growth periods, rather than as fundamental differences in bone tissue microstructural organization. Consequently, we consider the noticeable differences in overall bone tissue organization, paired with differences in bone morphology, to be sufficient justification for recognizing these as independent species.

**Figure 3:**
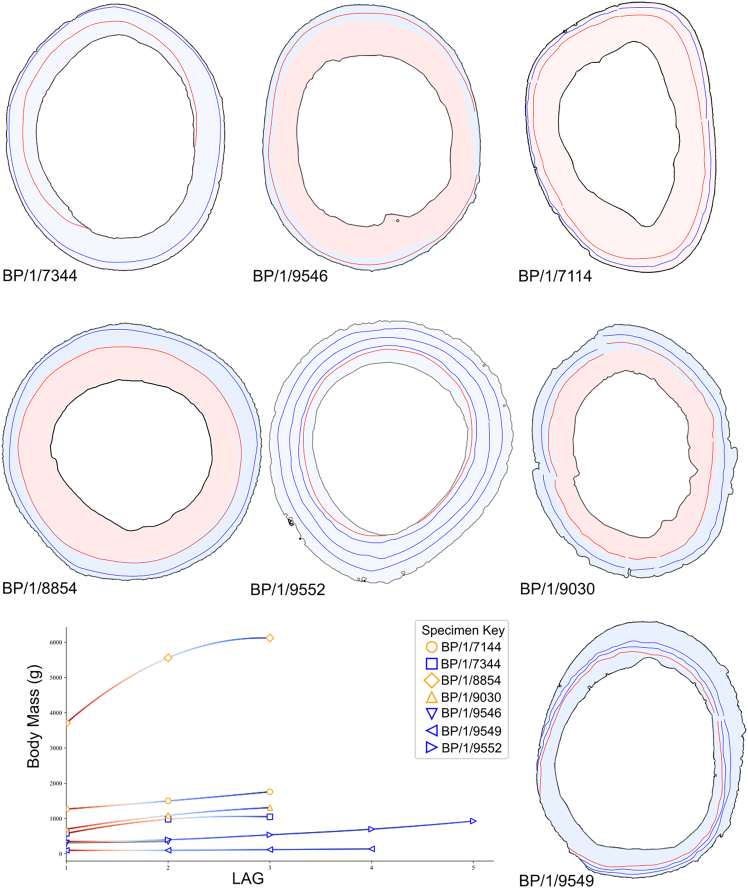
Cortical cross-sections of seven archosauromorph The humerus (BP/1/7114) and femora (BP/1/7344, BP/1/9546, BP/1/8854, BP/1/9552, BP/1/9030, and BP/1/9549); BP/1/6232 is excluded from the analysis because it shows no growth marks.The cross-sections show the medullary cavity in gray and growth marks (LAGs) outlined in either blue or red. The accompanying graph illustrates body mass increase across successive LAGs. Each curve represents an individual specimen, with line color corresponding to relative growth rate (calculated as body mass increase per LAG). Warmer colors (red) indicate more rapid growth; cooler colors (blue) indicate slower growth. LAGs on each cortical cross-section are color-matched to the corresponding growth rate. The outermost LAG in the graph represents the estimated body mass at the time of the final preserved bone circumference.

Importantly, the osteohistological variation documented here provides a window into the diversity of growth strategies among Early Triassic archosauromorphs (Figure 3). Our understanding of growth strategy diversity within in the larger archosauromorph tree remains limited, especially among its Triassic members. In particular, it is unclear whether the diversity observable in ornithodirans, and potentially earlier branching pseudosuchians, is an inherited trait from their shared archosauromorph ancestry. Previous investigations of stem archosauromorphs, such as *Proterosuchus*, *Erythrosuchus*, and *Garjainia*, showed that early archosauromorphs grew rapidly to at least mid-ontogeny, evidenced by the presence of a highly vascularized WPC throughout the cortex, even in subadults.^14^^,^^21^ These observations suggest that faster growth rates are potentially the plesiomorphic condition for the group.^22^ Other studies have shown a greater diversity in growth strategies among Triassic archosauromorphs in comparison to their later branching Jurassic counterparts, including slower-growing PFB and LB bone in *Trilophosaurus buettneri*^56^ and *Benggwigwishingasuchus eremacarminis*^22^ but these differences could be attributed to broad geographic dispersal,^22^ or in the case of aquatic tanystropheids, these growth strategies might reflect derived conditions necessitated by specialized modes of life, such as habitual submersion.

However, the Driefontein archosauromorph assemblage was recovered from a shared riparian environment within a localized geographic range, and although taxonomic diversity may reflect occupation of different ecological niches, there is insufficient variation in bone cortex thickness to indicate substantially different environmental conditions, such as an aquatic lifestyle. In the majority of the farm Driefontein 11 archosauromorphs examined, the presence of WPC is limited to the first growth season, after which there is a shift to predominantly slower depositing LB in individuals older than 1 year. In contrast, BP/1/7344, BP/1/9552, and BP/1/9549 show no evidence of an initial phase of rapid growth, instead forming only slower-depositing PFB or LB with more closely spaced LAGs throughout their documented period of development (Figure 3). These three specimens represent the smallest individuals in our sample, with maximum-known body masses close to or less than 1 kg. These observations could reflect a relationship between slower growth and smaller adult body sizes for some of the farm Driefontein 11 archosauromorphs when compared to *Garjainia*, or growth plasticity in response to ongoing stressed environmental conditions proposed for the Early Triassic,^57^ or, analogous to patterns seen in non-avialan theropods,^9^ a previously unrecognized diversity in growth strategies that hold no clear correlation to body size evolution.

However, regardless of the underlying cause, the results of this study clearly demonstrate that Early Triassic archosauromorphs exhibited a much broader range of growth strategies, and associated growth rates, than previously recognized. These observations support the hypothesis that faster and slower growth rates are not strongly linked to phylogeny among earlier branching Triassic archosaurs.^10^^,^^22^^,^^58^^,^^59^ Furthermore, although there is an overall trend crown-wards toward faster growth in avemetatarsalians and slower growth in crocodylomorphs, these growth strategies were not evolutionary innovations but instead represent end-members of variability in growth traits that were already ancestrally present among Triassic archosauromorphs.

### Limitations of the study

This study is based on a single Early Triassic archosauromorph assemblage from the Karoo Basin and therefore does not capture the full global taxonomic or growth strategy diversity of Early Triassic archosauromorphs. The material is highly fragmentary, permitting identification only to the clade Archosauromorpha and greatly limiting more precise phylogenetic interpretations. In addition, the sampled limb bones represent individuals with only partial life histories recorded. Although synchrotron micro-CT provides visualization of bone microstructure, some histological features remain difficult to resolve or interpret in samples that are greatly altered from diagenetic alternation or poor taphonomic preservation. Consequently, while our results demonstrate that variation in growth strategies was already present among Early Triassic archosauromorphs, broader evolutionary patterns will require expanded sampling from more completely preserved and taxonomically resolved fossil material across a wider phylogenetic range.

## Resource availability

### Lead contact

Further information and requests for resources should be directed to and will be fulfilled by the lead contact, Kathleen N. Dollman (dollman@esrf.fr).

### Materials availability

The fossils investigated in this study are housed at the Evolutionary Studies Institute, University of the Witwatersrand.

### Data and code availability

• All PPC-SRμCT data and virtual osteohistological thin sections have been deposited on the ESRF Paleontology Database (https://paleo.esrf.fr/) and are publicly available as of the date of publication. Accession numbers are listed in the key resources table, with a reference https://doi.org/10.15151/ESRF-DC-2379011853.
• This paper does not report original code.
• Any additional information required to reanalyze the data reported in this paper is available from the lead contact on request.

## Acknowledgments

The 10.13039/501100001671European Synchrotron Radiation Facility is thanked for the access to BM05 and BM18 for imaging of the samples. The staff on BM05 and BM18 are thanked for their assistance. We would like to especially thank Dr. Vincent Fernandez, Dr. Paul Tafforeau, and Prof. Sophie Sanchez for their assistance and many discussions on this project. J.B. and J.N.C. were funded by GENUS: DSTI-NRF Centre of Excellence in Palaeosciences, the 10.13039/501100001321National Research Foundation and the 10.13039/100016207Palaeontological Scientific Trust. The authors thank the editor and the anonymous reviewers for their constructive comments and suggestions, which significantly improved the manuscript.

## Author contributions

Conceptualization, K.N.D., F.B.T., J.N.C., A.B.H., V.B., J.B.; fossil collection, J.H.; synchrotron imaging, K.N.D. and C.M.; osteohistological analysis, K.N.D., J.N.C., and J.B.; morphological description, F.B.T and J.N.C.; writing, K.N.D.; F.B.T.; J.N.C. J.H., A.B.H., V.B., and J.B.

## Declaration of interests

The authors declare no competing interests.

## STAR★Methods

### Key resources table

**Table:** 

REAGENT or RESOURCE	SOURCE	IDENTIFIER
**Deposited data**		

2.53um_archosaurhisto_BP_1_5661	This paper	https://doi.org/10.15151/ESRF-DC-2379011853
2.53um_archosaurhisto_BP_1_6232	This paper	https://doi.org/10.15151/ESRF-DC-2379011853
HA900_0.72um_Archosaurhisto_BP6232_ROI1_v1	This paper	https://doi.org/10.15151/ESRF-DC-2379011853
360_archosaurhisto_BP_1_7114_femur	This paper	https://doi.org/10.15151/ESRF-DC-2379011853
HA900_0.72um_Archosaurhisto_BP1_7114_ROI1_v1	This paper	https://doi.org/10.15151/ESRF-DC-2379011853
2.53um_archosaurhisto_BP_1_9030	This paper	https://doi.org/10.15151/ESRF-DC-2379011853
2.53um_archosaurhisto_BP_1_8854	This paper	https://doi.org/10.15151/ESRF-DC-2379011853
2.53um_archosaurhisto_P297	This paper	https://doi.org/10.15151/ESRF-DC-2379011853
HA900_0.72um_archohisto_BP9546_ROI1_v1	This paper	https://doi.org/10.15151/ESRF-DC-2379011853
2.53um_archosaurhisto_BP_1_7344	This paper	https://doi.org/10.15151/ESRF-DC-2379011853
HA900_0.72um_archohisto_BP7344_ROI1_v1	This paper	https://doi.org/10.15151/ESRF-DC-2379011853
HA900_2um_archosaurhisto_DSP012_femur	This paper	https://doi.org/10.15151/ESRF-DC-2379011853
HA900_0.72um_Archosaurhisto_DSP012_femur_ROI1_v1	This paper	https://doi.org/10.15151/ESRF-DC-2379011853
360_archosaurhisto_N777_femur	This paper	https://doi.org/10.15151/ESRF-DC-2379011853
HA900_0.72um_Archosaurhisto_N777_femur_ROI1_v1	This paper	https://doi.org/10.15151/ESRF-DC-2379011853
2.53um_archosaurhisto_BP_1_5661	This paper	https://doi.org/10.15151/ESRF-DC-2379011853
2.53um_archosaurhisto_BP_1_6232	This paper	https://doi.org/10.15151/ESRF-DC-2379011853
HA900_0.72um_Archosaurhisto_BP6232_ROI1_v1	This paper	https://doi.org/10.15151/ESRF-DC-2379011853
360_archosaurhisto_BP_1_7114_femur	This paper	https://doi.org/10.15151/ESRF-DC-2379011853
HA900_0.72um_Archosaurhisto_BP1_7114_ROI1_v1	This paper	https://doi.org/10.15151/ESRF-DC-2379011853
2.53um_archosaurhisto_BP_1_9030	This paper	https://doi.org/10.15151/ESRF-DC-2379011853
2.53um_archosaurhisto_BP_1_8854	This paper	https://doi.org/10.15151/ESRF-DC-2379011853
2.53um_archosaurhisto_P297	This paper	https://doi.org/10.15151/ESRF-DC-2379011853
HA900_0.72um_archohisto_BP9546_ROI1_v1	This paper	https://doi.org/10.15151/ESRF-DC-2379011853
2.53um_archosaurhisto_BP_1_7344	This paper	https://doi.org/10.15151/ESRF-DC-2379011853
HA900_0.72um_archohisto_BP7344_ROI1_v1	This paper	https://doi.org/10.15151/ESRF-DC-2379011853
HA900_2um_archosaurhisto_DSP012_femur	This paper	https://doi.org/10.15151/ESRF-DC-2379011853
HA900_0.72um_Archosaurhisto_DSP012_femur_ROI1_v1	This paper	https://doi.org/10.15151/ESRF-DC-2379011853
360_archosaurhisto_N777_femur	This paper	https://doi.org/10.15151/ESRF-DC-2379011853
HA900_0.72um_Archosaurhisto_N777_femur_ROI1_v1	This paper	https://doi.org/10.15151/ESRF-DC-2379011853

**Other**		

Archosauromorph fossil complete humerus	Evolutionary Studies Institute, University of Witwatersrand	BP/1/6232
Archosauromorph fossil complete humerus	Evolutionary Studies Institute, University of Witwatersrand	BP/1/7114
Archosauromorph fossil complete ulna indet.	Evolutionary Studies Institute, University of Witwatersrand	BP/1/5661
Archosauromorph fossil proximal femur	Evolutionary Studies Institute, University of Witwatersrand	BP/1/8854
Archosauromorph fossil proximal femur	Evolutionary Studies Institute, University of Witwatersrand	BP/1/9546
Archosauromorph fossil complete femur	Evolutionary Studies Institute, University of Witwatersrand	BP/1/7344
Archosauromorph fossil distal femur	Evolutionary Studies Institute, University of Witwatersrand	BP/1/9552
Archosauromorph fossil proximal femur	Evolutionary Studies Institute, University of Witwatersrand	BP/1/9549
Archosauromorph fossil complete femur	Evolutionary Studies Institute, University of Witwatersrand	BP/1/9030

### Method details

#### Body mass estimate and growth curves

We studied ten limb bones, consisting of two humeri, six femora, an ulna and a fibula. The fossil material is fragmentary, with the majority of the limb bones preserving only the proximal or distal ends (Table 1). Nine limb bones can be more confidently identified as archosauromorphs. The exception is the ulna (BP/1/5661), which has only been tentatively referred to Archosauromorpha based on its resemblance to *Prolacerta broomi*. Therefore, we provide detailed descriptions of eight of the nine limb bones here, while reserving BP/1/5661 for the Supplementary Information.

We used the scaling relationships presented by Campione and Evans^1^ to estimate body mass. Since we are investigating isolated limb elements, we calculated the lower and upper 95 percentile estimates for body mass from measurements taken from either the femur or humerus, depending on which element was preserved. Body mass estimates based on femoral circumference were calculated with a least-squares regression model log(BM) = 2.8479× log (CF) - 0.4587, which has an R^2^ value of 0.9794, where BM = Body Mass and CF = Femoral Circumference. Body mass estimates based on humeral circumference where calculated with a least-squares regression model log(BM) = 2.6861 × log (CH) - 0.1438, which has an R^2^ value of 0.9816, CH = Humeral Circumference. To calculate growth parameters we measured the circumference of preserved LAGs following the methods outlined by Ricqlès et al.^12^^,^^13^ The medullary cavity and LAGs were measured using the polygon tool in ImageJ 2.16.0.^60^

#### X-ray tomography

We scanned the archosaur limb bones at beamlines BM05 and BM18 at the European Synchrotron Radiation Facility (ESRF) in Grenoble, France (https://doi.org/10.15151/ESRF-DC-2379011853). We imaged using propagation phase contrast synchrotron radiation micro-Computed Tomography (PPC-SRμCT). Samples were measured either at an isotropic voxel size of 2.53 μm (BM18), 2.02 μm (BM05) or 0.72 μm (BM05). For both experiments the synchrotron current was 200 mA with a filtered white beam, but the beamline was independently set up for each configuration (Table S1 in supplementary information). On BM18, with an isotropic voxel size of 2.53 μm, the measurements were taken at 160 keV with 3.75 mm of molybdenum attenuator, with a sample-to-detector propagation distance of 2 m^61^ and a 500 μm-thick LuAG scintillator. Data acquisition was carried out using an IRIS camera, capturing 6000 projections with a total exposure time of 150 ms with three accumulated subframes of 50 ms each.

On BM05, two beamline configurations were set up, but both acquisitions were taken with a PCO Edge 4.2 sCMOS camera (PCO, Kelheim, Germany), capturing 6000 projections. The first setup used an isotropic voxel size of 2.02 μm, with measurements taken at 97 keV with 1.24 mm of molybdenum attenuators, with a sample detector distance of 1.4 m and a 250 μm LuAg scintillator. The total exposure time for each acquisition was configured to 60 ms with three accumulated subframes of 20 ms each.^62^ The second configuration on beamline BM05 was at an isotropic voxel size of 0.72 μm. The measurements were taken at 97 keV with 0.92 mm of molybdenum attenuators, with a sample detector distance of 160 mm and a LSO24 scintillator. The total exposure time for each acquisition was configured to 100 ms. During acquisition on both BM05 and BM18 the center of rotation was offset to enlarge the field of view in the final reconstructed images. The final tomographic volume was reconstructed using PyHST2^63^ and processed with the single-distance Paganin phase retrieval.^64^ The osteohistological virtual thin sections were rendered in VG Studio Max 2024.1 (Volume Graphics, Heidelburg, Germany) using protocols developed by Smith et al.^65^ and Sanchez et al.*,*^34^ with transverse cross sections rendered as thick slabs with an average thick slab of 0.1 mm, a slice averaging of 1000, scale and oversampling of 1. The osteohistological description and identification of bone tissue follow the terminology de Buffrénil.^58^

### Quantification and statistical analysis

There are no quantification or statistical analyses to include in this study.
