# Supplementary material for: Synchrotron imaging reveals diversity in archosauromorph growth strategies by the end of the early Triassic
Source: iScience. 2026 Jun 3;29(6):116162. doi: 10.1016/j.isci.2026.116162 (PMC13255036; doi:10.1016/j.isci.2026.116162)
Supplement: Document S1. Figures S1–S10, Tables S1–S3, and Methods S1 [file mmc1.pdf]

iScience, Volume *29*

Supplemental information

Synchrotron imaging reveals diversity in archosauromorph growth strategies by the end of the early Triassic

Kathleen.N. Dollman, Frederick.B. Tolchard, Jonah.N. Choiniere, John Hancox, Andrew B. Heckert, Chandelé Montgomery, Valentin Buffa, and Jennifer Botha

Methods S1 Section: detailed description of morphology and identification of limb bones

A total of twelve limb bones were sampled for osteohistology for this study (Figure S1), of which ten were identified to at least a clade. There are two humeri considered in this sample: one large complete humerus (BP/1/6232) and one small fragmentary humeral head (BP/1/7114). They differ markedly in size and certain aspects of morphology. We, therefore, assume the specimens to represent two different taxa. Here, we provide a morphological description and overview of possible taxonomic affinities of each specimen.

BP/1/5661 (S1. A, B) is a zeugopod element as evidenced by its long, slender form and and concave proximal and distal articular surfaces. While difficult to diagnose, it is most likely an ulna based on the presence of an incipient olecranon process. In lieu of discrete characters supporting taxonomic identification, this specimen is referrable to Archosauromorpha based on a long, gracile morphology, being similar in proportion to appendicular elements of *Prolacerta broomi* (BP/1/2675). This shape is not similar to any cynodont alive at the time (e.g., *Langbergia modisei*; BP/1/5400, BP/1/5404). It is also considerably longer than any postcranial element from any known trirachodontid from the *Langbergia-Garjainia* Subzone (LGSz).

BP/1/7114 (S1. C, F) only comprises the proximal portion of a humerus. The humeral heads are mostly summetrical and the internal tuberosity is not markedly distinct from the humeral head, contrary to the condition in BP/1/6232. A very prominent conical process is situated dorsal to the deltopectoral crest (Character 420.1; Ezcurra 2016). This structure in BP/1/7114 closely resembles that of *Prolacerta broomi* (BP/1/2675), as well as that of the allokotosaurians *Malerisaurus* (Sengupta et al. 2024), *Pamelaria* (Ezcurra, 2016), and *Trilophosaurus* (Spielmann et al. 2008). Furthermore, there is a mediolaterally oriented groove along the proximal surface of the humerus of BP/1/7114, closely resembling *Prolacerta broomi* (BP/1/2675). A marked ridge runs over the dorsal surface of the bone, extending distomedially from the base of the deltopectoral cest to the distal end of the proximal epiphysis. Such a ridge is also present in Prolacerta (BP/1/2675), but this feature is absent in allokotosaurs[1,2]. While allokotosaur affinities are still possible based on the presence of the conical process, the presence of this ridge, as well as the overall size and dimensions of BP/1/7114 is more suggestive of prolacertid affinities.

BP/1/6232 (S1. D, E, G, H) is referred to Archosauromorpha because of: (a) the absence of an entepicondylar foramen (Character 423.1; Ezcurra 2016), and (b) an incipient ulnar condyle (trochlea; Character 429.1; Ezcurra 2016). The specimen also shows (a) a medially expanded proximal epiphysis giving the bone a slightly asymmetrical head, and (b) a medial tuberosity (= internal tuberosity) distinctly separated from the proximal articular surface by a notch. Ezcurra (2016; characters 419.1 and 421.1) recovered both features as synapomorphies of Archosauria. Furthermore, immediately medial to this notch, BP/1/6232 exhibits (c) a pointed

lip with a slightly concave proximal surface extending to the level of the midline of the proximal epiphysis. In these three features, the proximal humeral head of BP/1/6232 is exceedingly similar to those of *Prestosuchus chiniquensis*[3] and *Nundasuchus songeaensis*[4], which could suggest Pseudosuchian affinities for this specimen. Conversely, the dorsal margin of the deltopectoral crest is marked by a conspicuous tubercle, a feature otherwise unique to the erythrosuchid *Garjainia madiba*[5,6]. Also in common with *Garjainia madiba*, the deltopectoral crest is trapezoidal in lateral view (Ezcurra 2016; character 420.1). In addition, the distal epicondyles are clearly distinct, separated by the concave distal margin of the bone, and the entepicondyle extends slightly distal to the ectepicondyle and the humeral condyles. This morphology is more reminiscent of non-archosaurian allokotosaurs [7–9] or non-archosaurian archosauriforms[6,10]. In light of all this, BP/1/6232, we here conservatively refer BP/1/6232 to Archosauriformes.

There are six femora considered in this sample: two complete femora (BP/1/9030, S1. I, J; BP/1/7344, S1. K, L), three in which only the proximal portion is preserved (BP/1/8854, S1. M, N; BP/1/9546, S1. P; BP/1/9549, S1. Q), and one in which only the distal portion is preserved (BP/1/9552, S1. O). All specimens with preserved proximal portions are referable to the clade including Archosauriformes and several subclades of non-archosauriform archosauromorphs (unnamed node “tanystrophidae+archosauria”, sensu Ezcurra 2016) by the following synapomorphy: partially ossified proximal articular surface with a circular concavity (491.1; Ezcurra 2016). These femora can also be excluded from Eucrocopoda by the absence of a fourth trochanter (504.0; Ezcurra 2016). In each such instance, the proximal surface is sub-circular/crescent-shaped with both the anterior and posterior margins convex in proximal view. The internal trochanter is prominent and extends far medially from the level of the proximal surface. This morphology strongly reflects that of *Mesosuchus browni* (SAM-PK-7416), *Prolacerta broomi* (BP/1/2675) and *Proterosuchus* (SAM-PK-K140). Where preserved, all distal margins are sub-rectangular, with the distal epiphysis being markedly wide than the diaphysis, as is the case in *Prolacerta broomi* (BP/1/2675). The medial and lateral condyles are small protrusions, subtriangular in ventral view. The crista tibiofibularis presents as a small, pointed process directly posterior to the lateral condyle. This morphology closely resembles that of *Prolacerta broomi* (BP/1/2675), but also other early-branching archosauriforms such as *Proterosuchus* (SAM-PK-K140).

Methods S1 Section: detailed description of osteohistology

BP/1/5661 is identified as an ulna, has a thick cortex, with a wide, open medullary cavity (Figure S2). However, ulnae are known for having thicker cortices than either femora or humeri. Evidence of bone remodelling is present, with resorption cavities visible in the inner cortex. The vascular canals are primarily longitudinally oriented, with radial canals in some areas. The bone tissue is woven-parallel complex with the presence of primary osteons.

There is a change in intensity of primary osteons from the inner to outer cortex. The inner cortex is densely aggregated with primary osteons, with a reduction in intensity towards the outer cortex. The osteocyte lacunae are randomly distributed in the inner and mid-cortex and along with the abundant vascular canals. Approximately half way through the cortex the bone tissue transitions to a relatively slower forming woven-parallel-fibered or lamellar bone. There is a LAG in the inner cortex and another in the mid-cortex interrupting the rapidly forming bone tissue. There does not appear to be any further growth mark closer to the outer cortex. Given the presence of at least two growth marks and the overall decrease in growth rate towards the sub-periosteal surface, this individual was likely a subadult.

BP/1/6232 exhibits a thick cortex and with a large, open medullary cavity (Figure S3). The bone shows minimal secondary remodelling and is highly vascularized, with longitudinal canals and short anastomoses forming radiating rows and sub-reticular arrangements, suggesting rapid growth. The randomly oriented osteocyte lacunae, combined with the vascular pattern, indicate that the bone tissue is woven fibred bone tissue. The absence of growth marks and "bands" of decreased vascularization suggests that the individual was likely a juvenile at the time of death, and the thick cortical bone supports the idea that early growth marks were unlikely to have been erased.

BP/1/7114, a humerus, has a relatively thin and compact cortex, with a large, open medullary cavity (Figure S4.). The section was taken more proximally, revealing some trabeculae in the medullary cavity. The bone tissue is highly vascularised with simple, longitudinal canals. The inner to mid cortex comprises of woven – parallel fibre complex with large primary osteons present. Lamellar bone is absent from the inner and mid-cortex. There are two relatively closely spaced LAGs are present in the outer cortex. Although there is no change in bone tissue type in this region, the close spacing of the LAGs suggests that the amount of annual bone deposition had begun to decrease.

BP/1/9030, a femur, is characterized by a relatively thin, compact cortex surrounding a large, open medullary cavity (Figure S5). There is no indication of secondary remodelling. The cortex is composed of highly vascularized bone tissue from the inner to mid-cortex, transitioning to very poorly vascularized bone tissue in the outer third with the canals ranging from longitudinal to radial orientations. Numerous large, clustered osteocyte lacunae in the inner and mid-cortex indicate an initial faster growth rate which then slows down later in development. Multiple lamellae in the outer cortex represent individual bone lamellae and annual growth marks, though it is difficult to clearly distinguish individual growth marks. However, at least two growth marks appear to be present, with one traversing the more vascularized mid-cortex. This suggests that, after the first year of growth, the individual's growth rate began to slow. The presence of vascular canals in the outer cortex indicates that this outer slow-growing region likely does not represent an OCL, which is characterized by avascular, slow-

growing bone and may or may not include growth marks. However, the significant decline in growth rate by the time of death suggests that this individual was unlikely to grow much larger. Therefore, this specimen likely represents a small-bodied species, similar to BP/1/8854, but with some notable differences in growth patterns and bone structure.

This femur of ***BP/1/8854*** exhibits a relatively thin compact cortex and a large, open medullary cavity (Figure S6). From the mid-cortex to the sub-periosteal surface, there is a clear transition from woven parallel complex to lamellar bone. The inner cortex appears is composed of woven parallel complex, with randomly oriented osteocyte lacunae and primary osteons. No evidence of secondary remodelling, secondary osteons, or resorption cavities is present. The inner cortex contains longitudinally oriented vascular canals, arranged in radial rows, with some regions displaying short radial canals. In the outer cortex, the vascular canals generally appear simpler. Two growth marks are visible: one in the mid-cortex and another near the sub-periosteal surface. Although vascular canals extend to the outermost cortex, indicating that this individual was still growing at the time of death, the decrease in vascularization suggests that the growth rate had decreased. Some of the vascular canals in the outer cortex are surrounded by black “out-growths”. This feature looks similar to what is found in traditional thin sections and is usually attributed to some kind of diagenesis like fungal growth. The combination of a clear transition to slow-growing bone tissue and reduced vascularization strongly indicates that this individual had passed the juvenile stage and was likely a subadult of a small-bodied species at the time of death.

BP/1/9546 This section is taken from a femur of a small bodied archosaurmorph. with a femoral circumference of 11.32mm and an estimated body mass of 0.35kg. BP/1/9546 exhibits a relatively thin compact cortex with minimal observable remodelling, though two areas of resorption in the cross section are present (Figure S7). The low vascularization and presence of simple, longitudinally oriented canals indicates slow growth. There is a notable transition in tissue type from the inner to the outer cortex. The inner cortex is more densely populated with primary osteons. The random arrangement of osteocyte lacuna together with the absence of lamellae suggests a woven-parallel complex. The outer cortex shows greater organization, with visible lamellae indicative of lamellar or parallel-fibered bone tissue. A double LAG lines are indicative of a temporary cessation in growth was observed at the outer cortex. The specimen was one year old when it died, a juvenile. However, given the presence of an overall decrease in vascularisation towards the subperiosteal surface it is possible that the overall growth rate of this individual had begun to decrease.

BP/1/7344 is identified as the femur, displays a relatively thinner compact cortex in comparison to BP/1/8854. The measured femoral circumference is 16.69mm with an estimated body mass of 1.05kg. Evidence of resorption is present along the inner endosteal margin but no secondary osteons are observed (Figure S8). Small osteons are visible at 0.72µm, their thin flat osteocytes which are arranged parallel to the cortex of the bone indicates parallel

fibre bone tissue. This characterization is further supported by the low vascularization with simple, longitudinally oriented canals and the lack of secondary osteons. The presence of parallel fibre bone tissue with lamellae indicates a slow growth rate overall. Two growth marks are present with the inner growth mark representing a double LAG and the second growth mark situated at the subperiosteal surface. Despite the small size of the bone, this individual does not represent a juvenile as it was two years old at the time of death.

The cortex of ***BP/1/9552*** is relatively thin and compact, with a wide, open medullary cavity (Figure S9). The cortical surface and outermost periosteal region show signs of erosion, preventing confirmation of the presence of OCL, which would indicate essentially a cessation in growth. Measurements taken of the circumference of the femur (15.96mm) indicate a body mass of 0.93kg. Secondary remodelling was not observed. The vascular canals are simple and some of them are radially oriented, a pattern more commonly associated with lamellar bone tissue in birds and mammals, as seen in other taxa[13]. There is a shift in bone tissue from mid to outer cortex, the inner cortex is more vascularized with the presence of some reticular canal networks. Additionally, the inner cortex shows some primary osteons. From the mid to outer cortex the vascularization is more radially orientated with an absence of primary osteons. The outer edge of the cortex is porous, likely because of secondary processes like fungal activity, and so it is not possible to discern clearly the microstructures in these affected areas. The cortex is traversed by numerous lamellae. Osteocyte lacunae are oriented parallel to the lamellar bone matrix, further supporting the identification of the bone tissue as lamellar. At least four LAGs are present in the cortex, however, it is difficult to distinguish the lamellae of the lamellar bone from growth marks and it is possible there may be more growth marks. However, the presence of even four growth marks indicates this animal was not a juvenile, but a subadult at the time of death. The presence of lamellae throughout the cortex indicates slow growth, but the presence of some reticular vascularization indicates a faster relative growth rate earlier in ontogeny. Overall, given the mass, age and growth rate of this individual, this bone histology represents a small bodied, slow growing archosauromorph.

BP/1/9549 exhibits a very thin and compact bone cortex with a large, open medullary cavity, and no visible resorption cavities and devoid trabeculae (Figure S10). The femur is very small, the smallest of the sampled archosauromorphs, with a measure circumference of 8.21mm and estimated body mass of 0.14kg. A visible incision into the endosteum in the cross-section is attributed to a large vascular canal traversing the cortex. The vascularization is composed of simple, longitudinal canals that are randomly distributed throughout the cortex. Vascularization extends to the sub-periosteal region, indicating that this individual was still growing when it died. Primary osteons are observed throughout the cortex. The even distribution and orientation of the osteocyte lacunae indicates a parallel-fibered bone tissue, with lamellar regions where there are growth marks. There is no discernible shift in bone tissue type, suggesting that a relatively slow growth rate persisted throughout the individual's development. Multiple growth cycles are evident, with at least three Lines of Arrested Growth

(LAGs) present; a potential fourth LAG is observed, but it does not continue unbroken around the cortex. Given that this individual was at least three years old at the time of death and had not yet reached full growth, combined with a slow growth rate throughout development, it is unlikely that this individual would have achieved a much larger body size. It is therefore probable that this individual represents a slow-growing, small-bodied species.

Supplementary Tables

Collection number	File header	Beamline	Voxel size (µm)	Detected energy	Total exposure time	Propagation distance	Attenuators	Projections
**BP/1/5661**	2.53um_archosaurhisto_BP_1_5661	BM18	2.53	160	50ms acc3	2m	Mo 3.75mm	6000
**BP/1/6232**	2.53um_archosaurhisto_BP_1_6232	BM18	2.53	160	50ms acc3	2m	Mo 3.75mm	6000
**BP/1/6232**	HA900_0.72um_Archosaurhisto_BP6232_ROI1_v1	BM05	0.72	92	100ms acc1	160mm	Mo 0.92mm	6000
**BP/1/7114**	360_archosaurhisto_BP_1_7114_femur	BM05	2.02	97	20ms acc 3	1.4m	Mo 1.24 mm	6000
**BP/1/7114**	HA900_0.72um_Archosaurhisto_BP1_7114_ROI1_v1	BM05	0.72	92	100ms acc1	160mm	Mo 0.92mm	6000
**BP/1/9030**	2.53um_archosaurhisto_BP_1_9030	BM18	2.53	160	50ms acc3	2m	Mo 3.75mm	6000
**BP/1/8854**	2.53um_archosaurhisto_BP_1_8854	BM18	2.53	160	50ms acc3	2m	Mo 3.75mm	6000
**BP/1/9546**	2.53um_archosaurhisto_P297	BM18	2.53	160	50ms acc3	2m	Mo 3.75mm	6000
**BP/1/9546**	HA900_0.72um_archohisto_BP9546_ROI1_v1	BM05	0.72	92	100ms acc1	160mm	Mo 0.92mm	6000
**BP/1/7344**	2.53um_archosaurhisto_BP_1_7344	BM18	2.53	160	50ms acc3	2m	Mo 3.75mm	6000
**BP/1/7344**	HA900_0.72um_archohisto_BP7344_ROI1_v1	BM05	0.72	92	100ms acc1	160mm	Mo 0.92mm	6000
**BP/1/9552**	HA900_2um_archosaurhisto_DSP012_femur	BM05	2.02	97	20ms acc 3	1.4m	Mo 1.24 mm	6000
**BP/1/9552**	HA900_0.72um_Archosaurhisto_DSP012_femur_ROI1_v1	BM05	0.72	92	100ms acc1	160mm	Mo 0.92mm	6000
**BP/1/9549**	360_archosaurhisto_N777_femur	BM05	2.02	97	20ms acc 3	1.4m	Mo 1.24 mm	6000

**BP/1/9549**	HA900_0.72um_Archosaurhisto_N777_femur_ROI1_v1	BM05	0.72	92	100ms acc1	160mm	Mo 0.92mm	6000

Table S1. A table presenting the scan parameters for each specimen imaged on BM05 or BM18 at the European Synchrotron and Radiation Facility.

**Collection Number**	**Identification**	**Maximum Body Mass Estimate (kg)**	**LAGs**	**Bone Tissue Type 1**	**Thickness (mm)**	**Percentage of Total Cortex**	**Growth Period**	**Bone Tissue Type 2**	**Thickness (mm)**	**Percentage of Total Cortex**	**Growth Period**
**BP/1/6232**	Archosauromorpha	11.73	0	Woven	2.70	100.00	0-max (<1)	-	-	-	-
**BP/1/7114**	Archosauromorpha	1.76	2	Woven	1.02	100.00	0-max (<3)	-	-	-	-
**BP/1/5661**	Archosauromorpha	-	3	Woven	1.14	52.42	0-2	Woven -Parallel	1.05	48.50	2-max (<4)
**BP/1/8854**	Archosauromorpha	6.12	2	Woven	1.18	59.27	0-1	Lamellar	0.81	40.73	1-max (<3)
**BP/1/9546**	Archosauromorpha	0.35	1	Woven	0.50	72.54	0-1	Woven-Parallel	0.19	27.46	1-max (<2)
**BP/1/7344**	Archosauromorpha	1.05	2	Parallel	5.98	100.00	0-max (<3)	-	-	-	-
**BP/1/9552**	Archosauromorpha	0.93	4	Lamellar	0.92	100.00	0-max (<5)	-	-	-	-
**BP/1/9549**	Archosauromorpha	0.14	3	Parallel	0.41	100.00	0-max (<4)	-	-	-	-
**BP/1/9030**	Archosauromorpha	1.31	2	Woven	1.08	100.00	0-max (<3)	-	-	-	-

Table S2. Table presenting a summary of changes in bone tissue types through development including the thickness and percentage contribution of the bone tissue type to the cortex in association with the LAG lines.

Collection Number	**Limb Element**	**Number of LAGs**	**LAG 1 circ.**	**LAG 1 estimated body mass**	**LAG 2 circ.**	**LAG 2 estimated body mass**	**LAG 3 circ.**	**LAG 3 estimated body mass**	**LAG 4 circ.**	**LAG 4 estimated body mass**	**Outer Circumference Measurement**	**Maximum estimated body mass (kg)**
**BP/1/7114**	Humerus	2	16.17	1.27	17.21	1.5	-	-	-	-	18.26	1.76
**BP/1/8854**	Femur	2	25.96	3.71	29.92						30.958	6.12
**BP/1/9546**	Femur	1	10.87	0.31	-	-	-	-	-	-	11.32	0.35
**BP/1/7344**	Femur	2	13.52	0.58	16.27	0.98	-	-	-	-	16.69	1.05
**BP/1/9552**	Femur	4	11.28	0.35	11.85	0.40	13.23	0.54	14.48	0.70	15.96	0.93
**BP/1/9549**	Femur	3	7.2	0.10	7.35	0.10	7.68	0.12	-	-	8.21	0.14
**BP/1/9030**	Femur	2	14.4	0.70	16.88	1.09	-	-	-	-	18.03	1.31

Table S3. Table presenting a summary increase in body measured from LAG lines in archosauromorph humeri for femora from Driefontein Farm 11.

Supplementary Figures

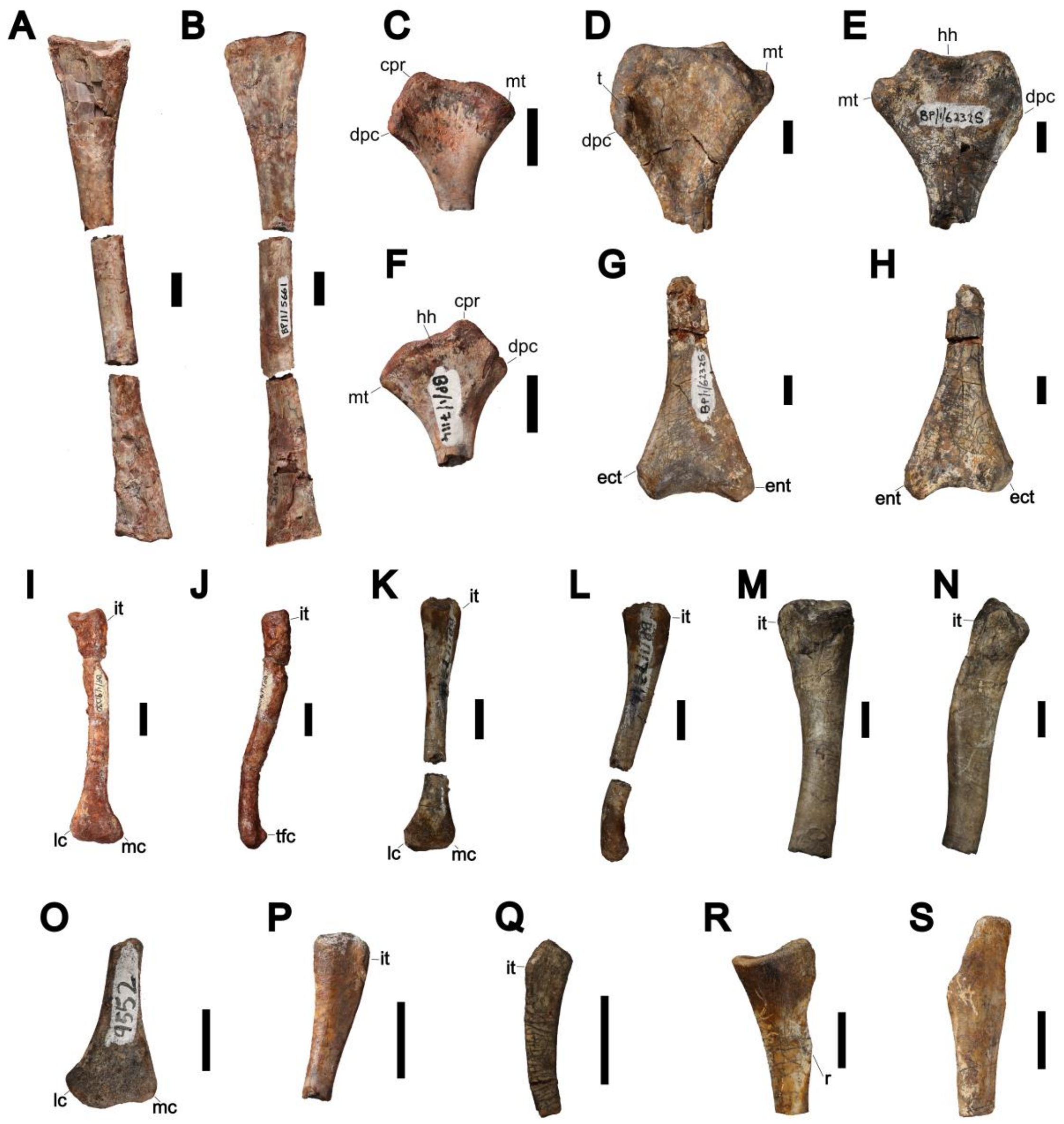

Figure S1. Postcranial elements of tetrapod fauna from site Driefontein 11. BP/1/5661, archoauromorph zeugopod element (A, B); BP/1/7114, archosauromorph proximal humerus, in ventral (C), and dorsal (F) views; BP/1/6232,

archosauriform humerus, proximal portion in lateral (D), and medial (E) views; BP/1/6232, archosauriform humerus, in distal portion in dorsal (G), and ventral (H) views; BP/1/9030, archosauromorph femur, in dorsal (I), and medial (J) views; BP/1/7114, archosauromorph femur, in dorsal (K), and medial (L) views; BP/1/8854, archosauromorph proximal femur, in dorsal (M), and medial (N) views; BP/1/9552, archosauromorph distal femur, in dorsal view (O); BP/1/9552, archosauromorph proximal femur, in medial view (P); BP/1/9549 archosauromorph proximal femur, in medial view (Q); BP/1/9554, proximal cynodont radius, in lateral view (R); BP/1/9555, fragment of cynodont radius shaft, in anterior view (S). **Abbreviations**: **cpr**, conical process; **dpc**, deltopectoral crest; **ect**, ectepicondye; **ent**, entepicondyle; **hh**, humeral head; **it**, internal tuberosity; **lc**, lateral condyle; **mc**, medial condyle; **mt**, medial tuberosity; **r**, ridge; **tfc**, tibiofibular crest. Scale bars represent 1 cm.

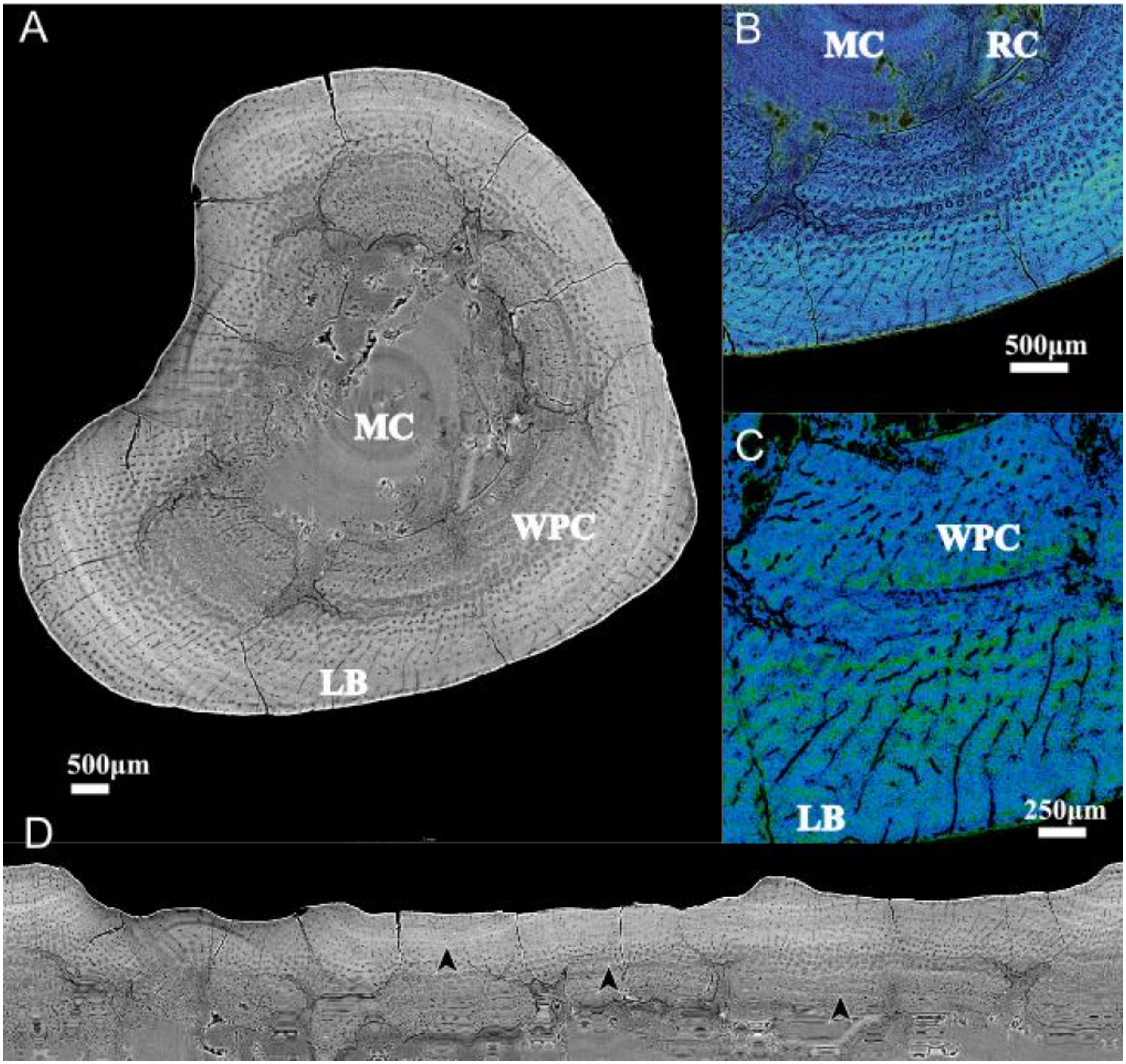

Figure S2. Bone tissue microstructure of BP/1/5661, A. a 2µm rendering of the complete cross section showing the bone tissue microstructures with B. optimized coloured histogram to contrast growth lines and osteons. C. A magnified section of a region on the cortex. D. the 2µm cross section rendering straight with red arrows indicating growth lines. Scale bars represent**:** 500 µm in A and B; and 250 µm in E.

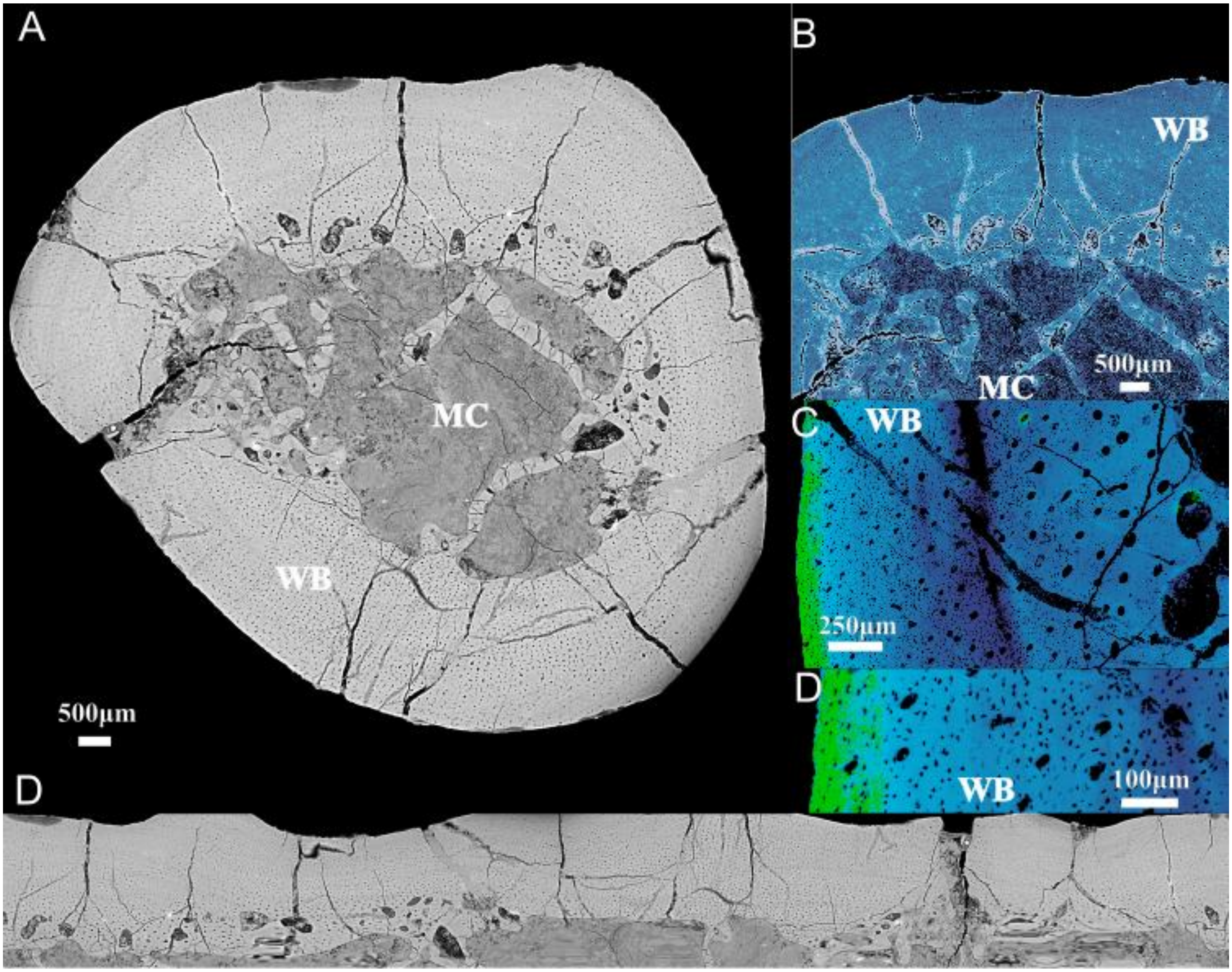

S3. Bone tissue microstructure of BP/1/6232, A. a 2µm rendering of the complete cross section showing the bone tissue microstructures with B. optimized coloured histogram of a region of 2µm scan to contrast growth lines and osteons. C. A rendering of a section of the cortex imaged at 0.72µm showing random osteocyte orientation and primary osteons. D. A close-up of a portion of the cortex at 0.72µm. E. the 2µm cross section rendering straight with red arrows indicating growth lines. Scale bars represent**:** 500 µm in A and B; 250 µm in C; and 100 µm in D.

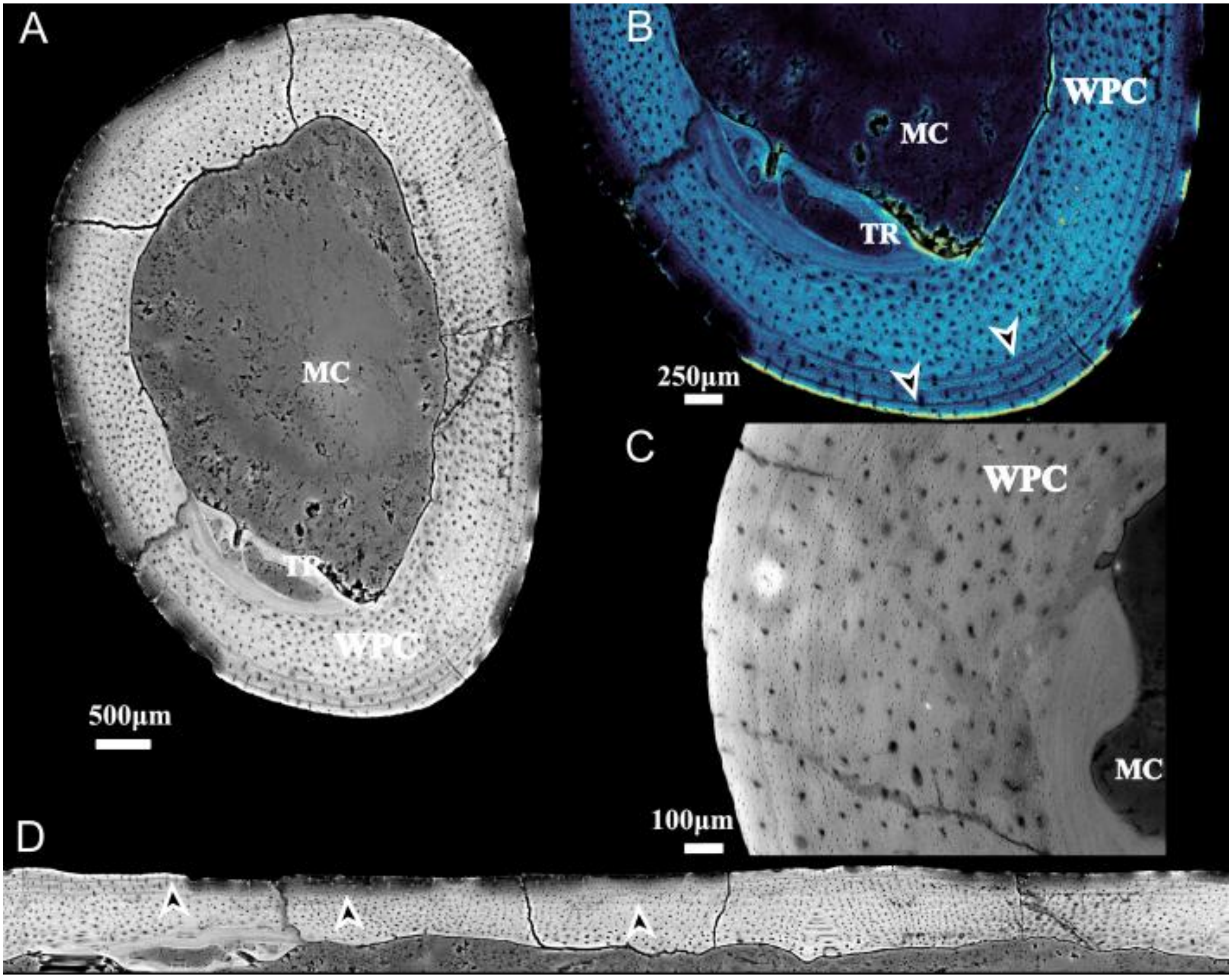

Figure S4. Bone tissue microstructure of BP/1/7114, A. a 2µm rendering of the complete cross section showing the bone tissue microstructures with B. optimized coloured histogram to contrast growth lines and osteons. C. A rendering of a section of the cortex imaged at 0.72µm showing random osteocyte orientation and primary osteons. D. the 2µm cross section rendering straight with red arrows indicating growth lines. Scale bars represent**:** 500 µm in A; 250 µm in B; and 100 µm in C.

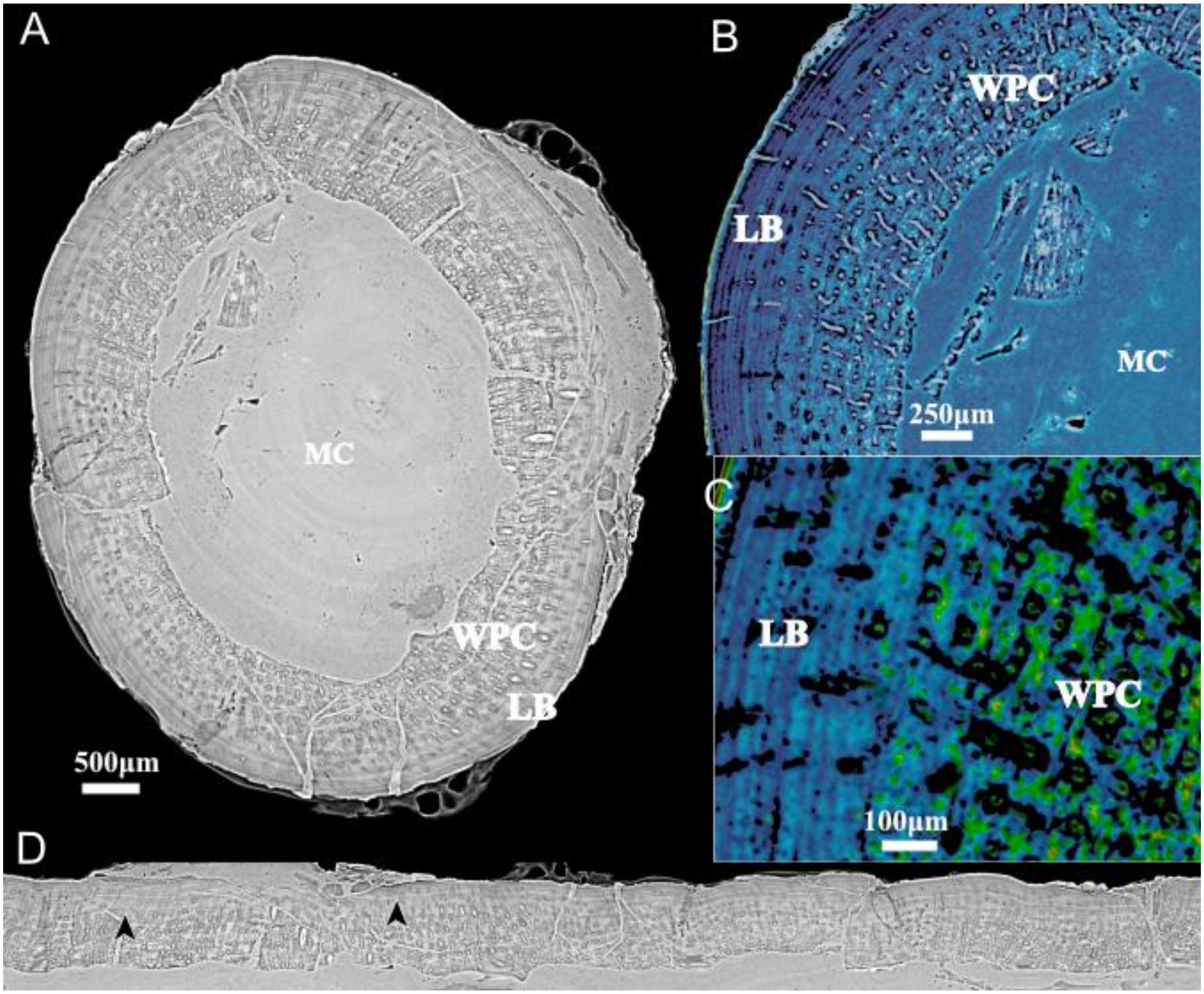

Figure S5. Bone tissue microstructure of BP/1/9030, A. a 2µm rendering of the complete cross section showing the bone tissue microstructures with B. optimized coloured histogram to contrast growth lines and osteons. C. A magnified section of a region on the cortex showing a shift in bone tissue type. D. the 2µm cross section rendering straight with red arrows indicating growth lines. Scale bars represent**:** 500 µm in A; 250 µm in B; and 100 µm in C.

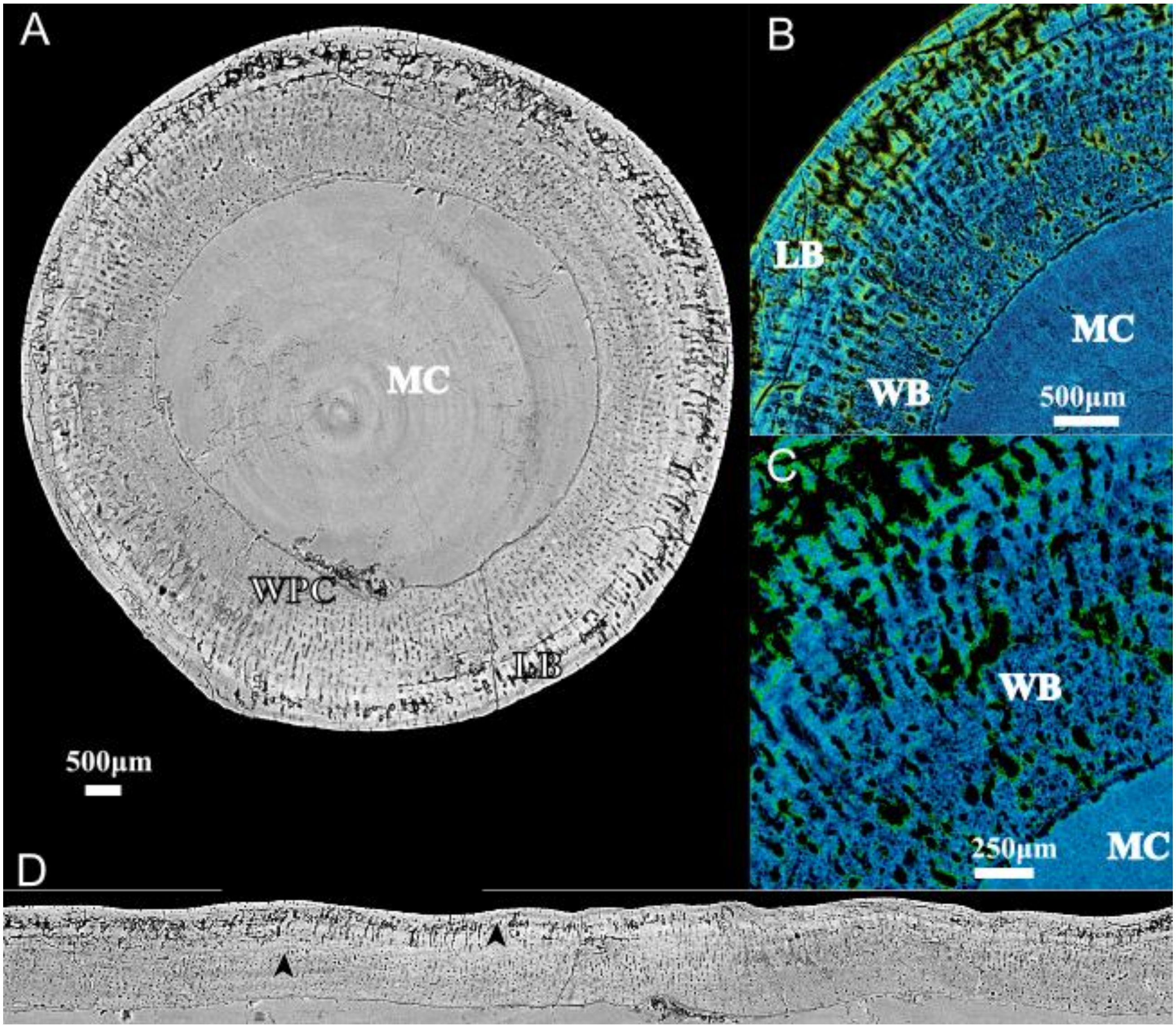

Figure S6. Bone tissue microstructure of BP/1/8854, A. a 2µm rendering of the complete cross section showing the bone tissue microstructures with B. optimized colour histogram to enhance contrast of growth lines and osteons. C. A magnified section of a region on the cortex. D. the 2µm cross section rendered straight with red arrows indicating growth lines. Scale bars represent**:** 500 µm in A and B; and 250 µm in C.

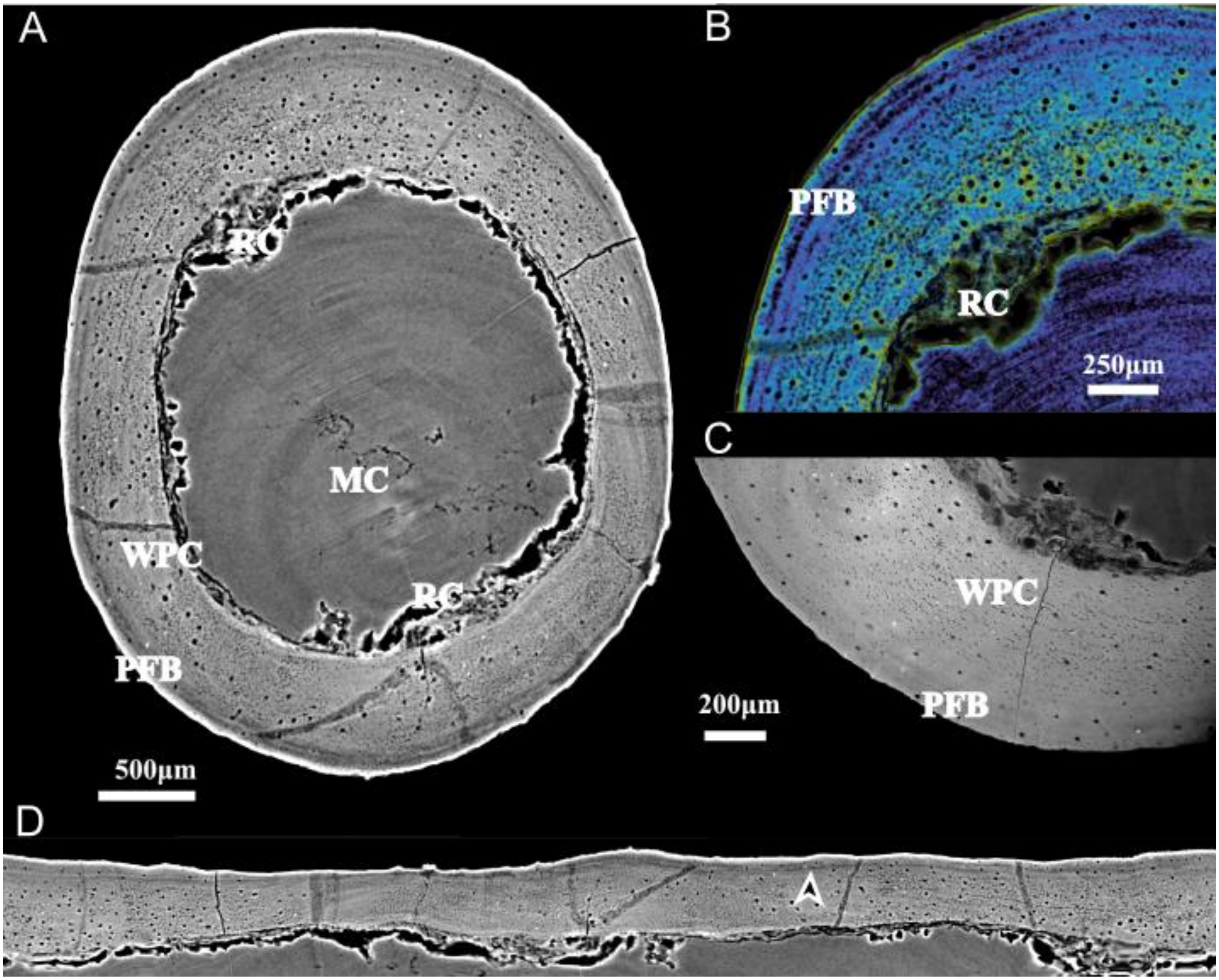

Figure S7. Bone tissue microstructure of BP/1/9546, A. a 2µm rendering of the complete cross section showing the bone tissue microstructures with B. optimized colour histogram to enhance contrast of growth lines and osteons. C. A magnified section of a region on the cortex rendered with an optimized grey value histogram. D. the 2µm cross section rendered straight with red arrows indicating growth lines. Scale bars represent**:** 500 µm in A; 250 µm in B; and 200 µm in C.

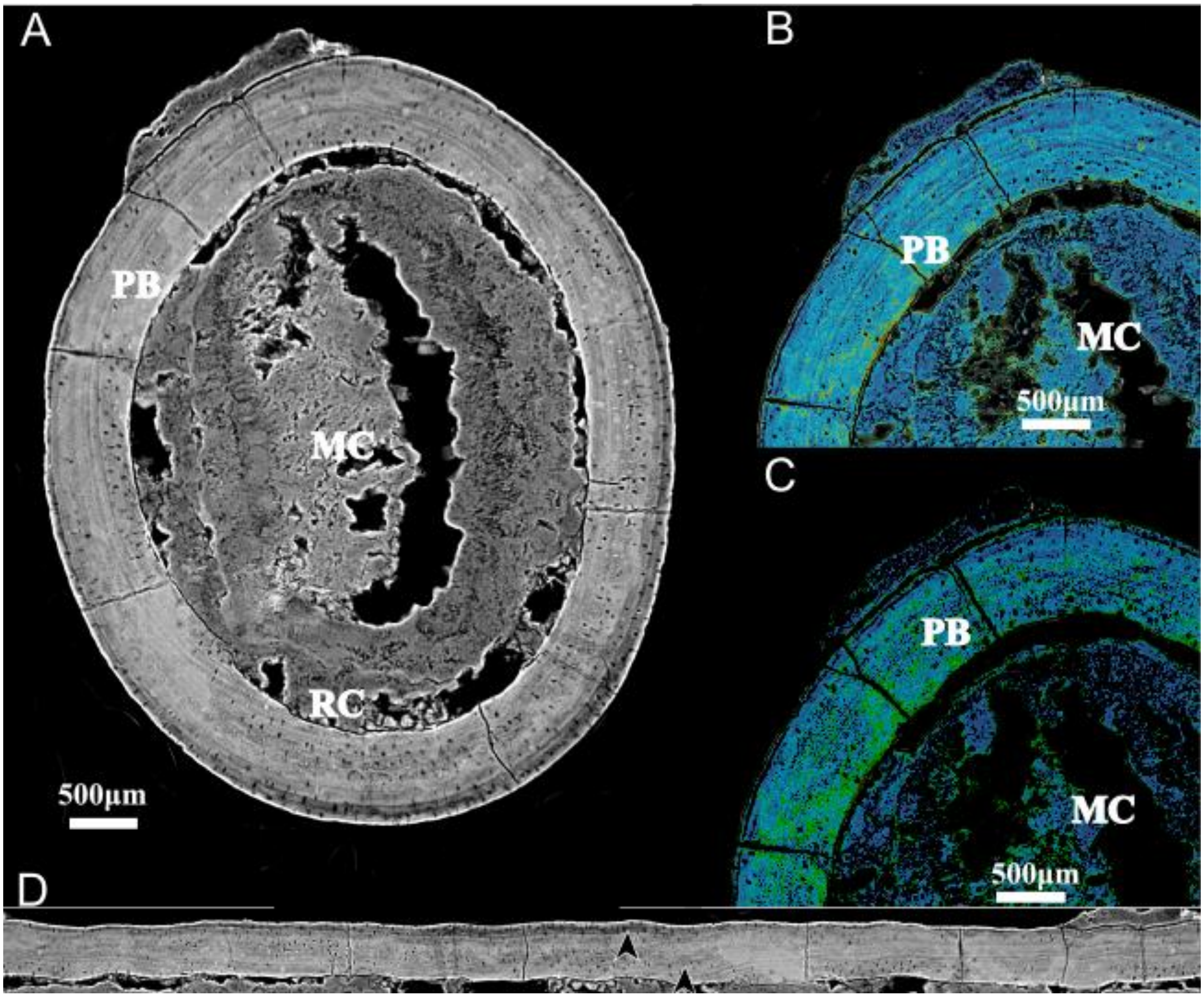

Figure S8. Bone tissue microstructure of BP/1/7344, A. a 2µm rendering of the complete cross section showing the bone tissue microstructures with B. optimized colour histogram to enhance contrast of growth lines and osteons. C. A rendering of a section of the cortex imaged at 0.72µm showing random osteocyte orientation and primary osteons. D. the 2µm cross section rendered straight with red arrows indicating growth lines. Scale bars represent**:** 500 µm in A, B and C.

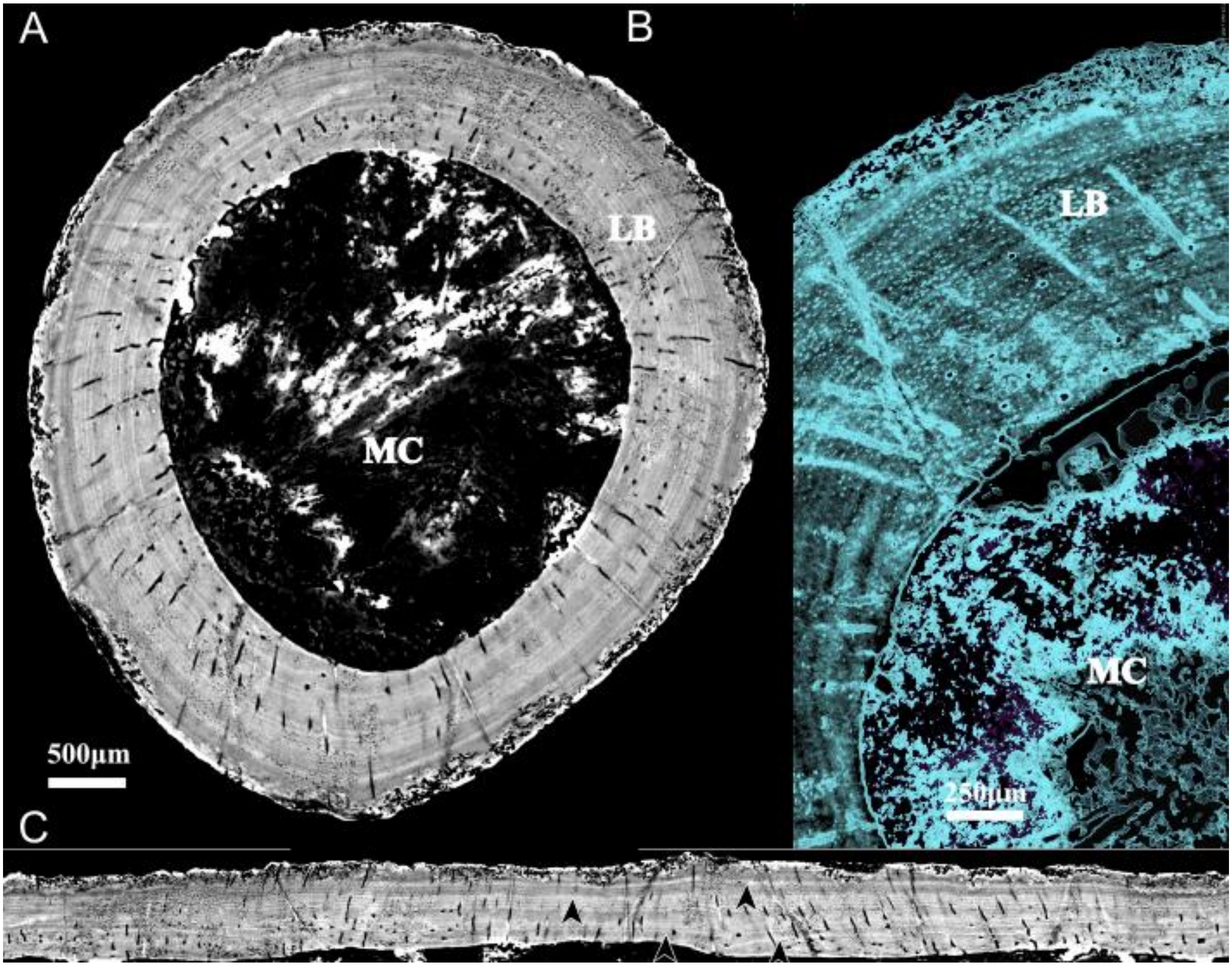

Figure S9. Bone tissue microstructure of BP/1/9552, A. a 2µm rendering of the complete cross section showing the bone tissue microstructures with B. optimized colour histogram to enhance contrast of growth lines and osteons. C. A rendering of a section of the cortex imaged at 0.72µm showing parallel fibre bone, parallel orientated osteocytes and primary osteons. D. the 2µm cross section rendered straight with red arrows indicating growth lines. Scale bars represent**:** 500 µm in A; and 250 µm in B.

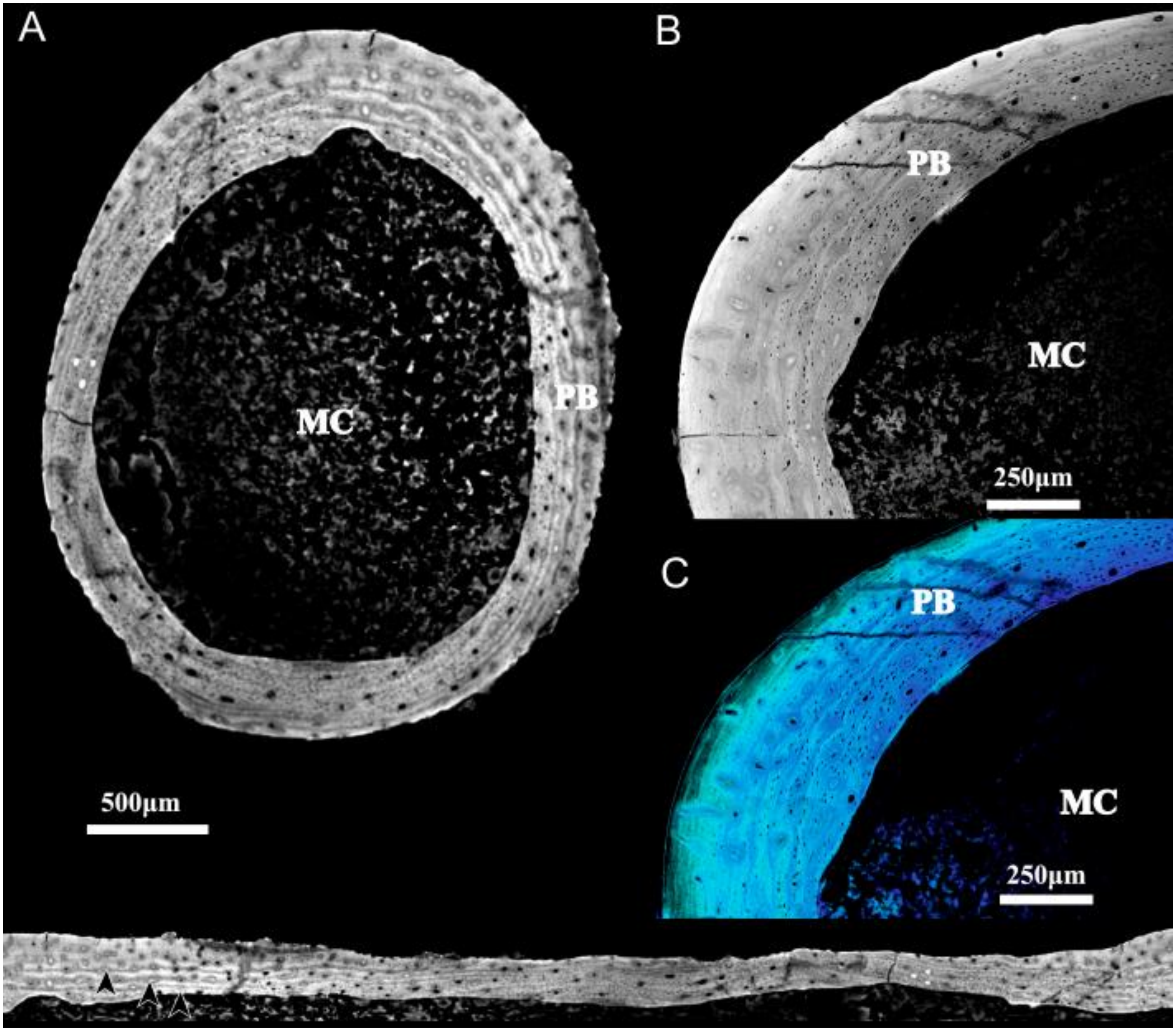

Figure S10. Bone tissue microstructure of BP/1/9549, A. a 2µm rendering of the complete cross section showing the bone tissue microstructures with B. A rendering of a section of the cortex imaged at 0.72µm showing parallel fibre bone, parallel orientated osteocytes and primary osteons, also rendered in C. with optimized colour histogram to enhance contrast of growth lines and osteons. D. the 2µm cross section rendered straight with red arrows indicating growth lines. Scale bars represent**:** 500 µm in A K; 250 µm in B and C.

Supplementary Information References

1. Spielmann, J. A., Lucas, S. G., Rhinehart, L. F. & Heckert, A. B. *The Late Triassic Archosauromorph Trilophosaurus: Bulletin 43*. vol. 43 (New Mexico Museum of Natural History and Science, 2008).
2. Nesbitt, S. J., Stocker, M. R., Chatterjee, S., Horner, J. R. & Goodwin, M. B. A remarkable group of thick-headed Triassic Period archosauromorphs with a wide, possibly Pangean distribution. *Journal of Anatomy* **239**, 184–206 (2021).
3. Mastrantonio, B. M. *et al.* Postcranial anatomy of Prestosuchus chiniquensis (Archosauria: Loricata) from the Triassic of Brazil. *The Anatomical Record* **307**, 925–956 (2024).
4. Nesbitt, S. J., Sidor, C. A., Angielczyk, K. D., Smith, R. M. & Tsuji, L. A. A new archosaur from the Manda beds (Anisian, Middle Triassic) of southern Tanzania and its implications for character state optimizations at Archosauria and Pseudosuchia. *Journal of Vertebrate Paleontology* **34**, 1357–1382 (2014).
5. Gower, D. J., Hancox, P. J., Botha-Brink, J., Sennikov, A. G. & Butler, R. J. A New Species of Garjainia Ochev, 1958 (Diapsida: Archosauriformes: Erythrosuchidae) from the Early Triassic of South Africa. in *PLOS ONE* vol. 9 1–35 (Public Library of Science, 2014).
6. Maidment, S. C. *et al.* The postcranial skeleton of the erythrosuchid archosauriform Garjainia prima from the Early Triassic of European Russia. *Royal Society Open Science* **7**, 201089 (2020).
7. Nesbitt, S. J. *et al.* Widespread azendohsaurids (Archosauromorpha, Allokotosauria) from the Late Triassic of western USA and India. *Papers in Palaeontology* **8**, e1413 (2022).
8. Sengupta, S., Ezcurra, M. D. & Bandyopadhyay, S. The redescription of Malerisaurus robinsonae (Archosauromorpha: Allokotosauria) from the Upper Triassic lower Maleri Formation, Pranhita-Godavari Basin, India. *The Anatomical Record* **307**, 1315–1365 (2024).
9. Marsh, A. D., Parker, W. G., Nesbitt, S. J., Kligman, B. T. & Stocker, M. R. Puercosuchus traverorum n. gen. n. sp.: a new malerisaurine azendohsaurid (Archosauromorpha: Allokotosauria) from two monodominant bonebeds in the Chinle Formation (Upper Triassic, Norian) of Arizona. *Journal of Paleontology* **96**, 1–39 (2022).
10. Sookias, R. B., Sullivan, C., Liu, J. & Butler, R. J. Systematics of putative euparkeriids (Diapsida: Archosauriformes) from the Triassic of China. *PeerJ* **2**, e658 (2014).
11. Gaetano, L. C., Mocke, H. & Abdala, F. The postcranial anatomy of Diademodon tetragonus (Cynodontia, Cynognathia). *Journal of Vertebrate Paleontology* **38**, e1451872 (2018).
12. Hancox, P. J., Neveling, J. & Rubidge, B. S. Biostratigraphy of the Cynognathus Assemblage Zone (Beaufort Group, Karoo Supergroup), South Africa. *South African Journal of Geology* **123**, 217–238 (2020).

13. de Margerie, E., Cubo, J. & Castanet, J. Bone typology and growth rate: testing and quantifying ‘Amprino’s rule’in the mallard (Anas platyrhynchos). *Comptes rendus biologies* **325**, 221–230 (2002).
